# Inhibition of Notch signaling rescues cardiovascular development in Kabuki Syndrome

**DOI:** 10.1371/journal.pbio.3000087

**Published:** 2019-09-03

**Authors:** Maria de los Angeles Serrano, Bradley L. Demarest, Tarlynn Tone-Pah-Hote, Martin Tristani-Firouzi, H. Joseph Yost

**Affiliations:** 1 Molecular Medicine Program—Neurobiology and Anatomy Department, University of Utah, Salt Lake City, Utah, United States of America; 2 University of Minnesota, Morris, Minnesota, United States of America; 3 Nora Eccles Harrison Cardiovascular Research and Training Institute and Division of Pediatric Cardiology, University of Utah, Salt Lake City, Utah, United States of America; University of Pennsylvania, UNITED STATES

## Abstract

Kabuki Syndrome patients have a spectrum of congenital disorders, including congenital heart defects, the primary determinant of mortality. Seventy percent of Kabuki Syndrome patients have mutations in the histone methyl-transferase *KMT2D*. However, the underlying mechanisms that drive these congenital disorders are unknown. Here, we generated and characterized zebrafish *kmt2d* null mutants that recapitulate the cardinal phenotypic features of Kabuki Syndrome, including microcephaly, palate defects, abnormal ear development, and cardiac defects. The cardiac phenotype consists of a previously unknown vasculogenesis defect that affects endocardium patterning and, consequently, heart ventricle lumen formation. Additionally, zebrafish *kmt2d* null mutants have angiogenesis defects depicted by abnormal aortic arch development, hyperactive ectopic blood vessel sprouting, and aberrant patterning of the brain vascular plexus. We demonstrate that zebrafish *kmt2d* null mutants have robust Notch signaling hyperactivation in endocardial and endothelial cells, including increased protein levels of the Notch transcription factor Rbpj. Our zebrafish Kabuki Syndrome model reveals a regulatory link between the Notch pathway and Kmt2d during endothelium and endocardium patterning and shows that pharmacological inhibition of Notch signaling rebalances Rbpj protein levels and rescues the cardiovascular phenotype by enhancing endothelial and endocardial cell proliferation and stabilizing endocardial patterning. Taken together, these findings demonstrate that Kmt2d regulates vasculogenesis and angiogenesis, provide evidence for interactions between Kmt2d and Notch signaling in Kabuki Syndrome, and suggest future directions for clinical research.

## Abstract 2

Kabuki Syndrome (KS) is a human congenital disorder with mutations in KMT2D. Zebrafish kmt2d mutants recapitulate human KS phenotypes, have aberrant Notch pathway signaling, and implicate vasculogenesis as a driver of abnormal cardiac development. Pharmacological inhibition of the Notch pathway rescues vasculogenesis in KS zebrafish, with potential therapeutic implications.

## Introduction

Kabuki Syndrome type I (KS, Online Mendelian Inheritance in Man [OMIM] number 147920) is a rare multisystemic disorder, manifested by craniofacial anomalies, including cleft lip and/or cleft palate and microcephaly, hearing loss, neurodevelopmental defects, epilepsy, skeletal and skin abnormalities, and congenital heart defects (CHDs) [1,2,11,3–10]. Although there is variable expressivity of the clinical hallmarks [8,12], CHD is present in approximately 70% of KS patients, with a unique predilection for left-sided obstructive lesions, including hypoplastic aortic arch, coarctation of the aorta, and hypoplastic left heart syndrome [13–17]. De novo pathogenic variants in Histone-lysine N-methyltransferase 2D (*KMT2D*) are causative in up to 76% of KS patients [18,19]. KMT2D, also known as MLL4 and MLL2 in humans and Mll2 in mice, belongs to a family of histone 3 lysine 4 methyltransferases [20], encodes a large protein with multiple domains, and plays critical roles regulating gene expression through epigenetics mechanisms. Despite great progress in genetic diagnosis for KS, our understanding of KS phenotypic variability and the downstream molecular pathways underlying the abnormal development of specific organ systems in KS is limited. Thus, characterizing the molecular mechanisms that drive phenotypic variation of KMT2D-dependent diseases is crucial for designing therapies to ameliorate these disorders.

Germline knockout of *Kmt2d* in mice is embryonic lethal, limiting the ability to model and study KS [21]. Conditional *Kmt2d* knockout in murine cardiac precursors and cardiomyocytes indicated that KMT2D is essential for regulating cardiac gene expression during heart development, primarily via di-methylation marks in lysine 4 of histone 3 (H3K4) [22]. The observation that cardiac progenitor-specific *Kmt2d* deletion mutants manifest more severe forms of CHD compared with myocardium-specific *Kmt2d* deletion suggests an unexplored critical role for Kmt2d other cardiovascular lineages, in particular in endocardium or endothelial lineages. Understanding the contribution of KMT2D to endocardial and endothelial development is crucial given that the prognosis of KS patients depends on the diagnosis and management of left-sided obstructive cardiovascular lesions [23].

Morpholino knockdown of *kmt2d* in zebrafish revealed craniofacial abnormalities, gross neurological defects, and anomalies in cardiac looping, suggesting that zebrafish might serve as a model for KS [24]. However, these phenotypes are common in morpholino treatments [25–27]. A recent study revealed a link between retrovirus-associated DNA sequence/Mitogen-activated protein kinase (RAS/MAPK) pathway hyperactivation and the neurological and craniofacial defects in the context of KS using *rap1a* and *rap1b* mutants and *rap1a*, *rap1b*, *raf1*, *kmt2d*, and *kmd6a* morpholino knockdowns in zebrafish [28]. In line with this, chemical inhibition of a downstream target of this pathway (v-raf murine sarcoma viral oncogene homolog B1 [BRAF] inhibitor), partially rescued the craniofacial and neuroanatomical phenotype of *kmt2d*-depleted zebrafish larvae in transient knockdown and *kmt2d*^*+/−*^ heterozygous crosses [29]. These findings suggest a pathway involved in some aspects of KS neurological defects and establishes the utility of zebrafish for drugs screening in KS. However, modeling cardiovascular developmental defects in KS and their molecular pathways, and possible approaches to reduce cardiovascular defects—the major cause of death in KS—have not been explored.

Although the molecular signatures driving KS phenotype have been reported [21,24,30], it remains unclear how KMT2D impacts cardiovascular patterning. Here, we generated zebrafish germline genetic mutants for *kmt2d* and validated them as models for KS by analyzing multiple cardinal clinical manifestations of this syndrome, including variable expressivity, short body length, palate defects, abnormal ear development, and heart defects. We identify for the first time a critical role for Kmt2d in vasculogenesis and angiogenesis and demonstrate a regulatory link between Kmt2d and Notch signaling. Moreover, pharmacological inhibition of Notch signaling rescues the cardiovascular defects observed in *kmt2d* mutant zebrafish, providing a platform for small molecule therapies to ameliorate the cardiovascular defects observed in KS patients.

## Results

### Zebrafish *kmt2d* null mutants exhibit phenotypes observed in human Kabuki Syndrome patients

Zebrafish *kmt2d* (ENSDARG00000037060) on Chromosome 23 contains 53 exons, with a 17.6 kb mRNA encoding a 362.7 KDa protein. Although the zebrafish Kmt2d protein (UniProt E7F2F7) only has 44.3% amino acid identity with the human KMT2D (UniProt O14686), BLAST analysis of the individual protein domains showed that the Plant Homeo Domain (PHD) located at the N terminus of the zebrafish Kmt2d has 86.1% identity with the human PHD (UniProt O14686: aa 5030 to 5137 and 126 to 217) and the Su-Enhancer-of-zeste and Trithorax (SET) and Post-SET domains located at the C terminus of the zebrafish protein have 99.1% and 100% identity with the human SET (O14686: aa 5398 to 5513) and Post-Set domains (O14686: aa 5521 to 5537), respectively. Important for functional analysis using reverse genetic approaches, this is the only ortholog for the human *Kmt2d* gene found in zebrafish, and there are no known paralogues.

**Fig 1:**
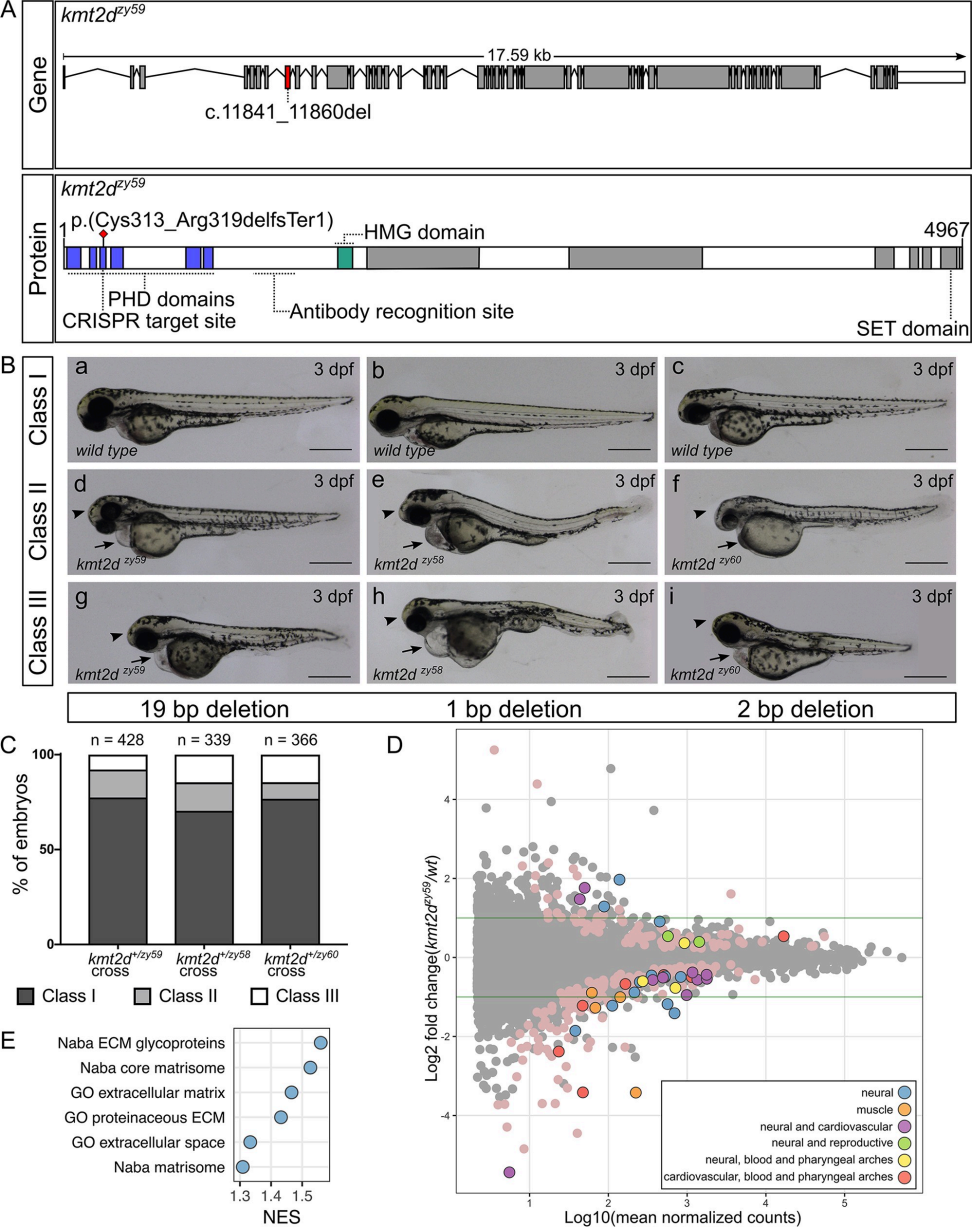
Generation and transcriptome profiling of *kmt2d* zebrafish mutants. (A) Schematic of zebrafish *kmt2d* gene showing the 19 bp deletion allele (*zy59*) and its predicted amino acid change in the protein. The gRNA was designed to target exon 8 (red shaded exon) at the 5ʹ end of the gene. The resulting 19 bp deletion is predicted to cause an early stop codon at the level of the PHD tandem domains (blue) located at the N terminus of the protein. The anti-Kmt2d antibody epitope is located after the PHD domains and before the HMG domain (green), allowing the validation of the early stop in *kmt2d*^*zy59*^ zebrafish null mutant. (B) Lateral views of zebrafish *wild-type* sibling embryos (a–c) and *kmt2d* mutants for 3 different alleles: *kmt2d*^*zy59*^ (a, d, g), *kmt2d*^*zy58*^ (b, e, h), and *kmt2d*^*zy60*^ (c, f, i) at 3 dpf. All 3 alleles shared the same phenotypic characteristics: microcephaly (arrowhead), heart edema (arrow), and mild to moderate body axis defects. Variable expressivity was observed in all the analyzed mutant alleles (Classes I to III). Scale bar = 500 μm. (C) Variable expressivity was analyzed in 3 different embryo clutches resulting from a heterozygous by heterozygous cross for each mutant allele. Embryos were ranked in 3 different classes based on the severity of the phenotype. Class I, *wild-type* and heterozygous siblings with no phenotype; class II, mutants with microcephaly and heart edema; class III, mutants with microcephaly, heart edema, and shorten body axes. Percentages of different clutches were calculated per the total number of living embryos for each genotype. Chi-square test (*p =* 0.14) and binomial test (*p =* 0.09) were performed to assess mendelian ratios considering heterozygous embryos within the Class I category. There was no significant discrepancy between obtained and expected percentages of embryo phenotype. Values for each data point can be found in S1 Data. (D) MA plot of differentially expressed genes from RNA-seq of individual *kmt2d*^*zy59*^ mutants (*n* = 6) versus *wild-type* (*n* = 6) sibling embryos at 1 dpf. The log_10_ of mean normalized counts are plotted against the log_2_ fold changes for each gene tested. Green horizontal lines represent 2-fold change differences. Negative log_2_ fold changes represent genes with reduced expression in the mutants relative to wild type. Both light pink points (no outline) and color-coded points (with outline) represent significantly differentially expressed genes (FDR adjusted *p* < 0.05). The top 50 genes (ranked by FDR adjusted *p*-value) were classified into 6 categories based on expression data, bibliography, phenotype information, and gene ontology (S1 Table). Raw data used for this analysis can be found in the following repository: https://b2b.hci.utah.edu/gnomex/gnomexFlex.jsp?requestNumber=475R. (E) GSEA. Analysis was performed by converting zebrafish gene names to human gene names using genes with a one-to-one ortholog relationship retrieved from Ensembl Compara database. The number of resulting genes identifiers analyzed was 9,128 out of 33,737 (Genome build Zv9, Ensembl annotation released version 79). Adjusted *p*-values were calculated per category. NES of gene sets with a FDR of 5% are plotted to summarized GSEA results. dpf, days post fertilization; ECM, extracellular matrix; FDR,; GO, gene ontology; GSEA, gene set enrichment analysis; HMG, High Mobility Group domain; Kmt2d, Histone-Lysine N-Methyltransferase 2D; NES, Normalized Enrichment Score; PHD, Plant Homeo Domain; RNA-seq, RNA sequencing; SET: Su-Enhancer-of-zeste and Trithorax domain.

To evaluate Kmt2d functional roles in zebrafish, we used CRISPR/Cas9 genome editing to generate zebrafish *kmt2d* null mutants. Exon 8 (ENSDARE00001117370), which contains the coding sequence for a PHD domain of the Kmt2d protein (Fig 1A), was targeted with a single guide RNA (sgRNA). On-target mutagenesis of injected embryos (F0) was confirmed by High Resolution Melt Analysis (HRMA) of the predicted target region for the sgRNA. Germline transmission of mutant alleles was confirmed by F1 genotyping. Genotyping of individual F1 adults revealed multiple *kmt2d* alleles, 3 of which were selected for propagation and additional analysis: *kmt2d*^*zy58*^ (Fig 1B [panels b, e, h; 1bp deletion]), *kmt2d*^*zy59*^ (Fig 1B [panels a, d, g; 19 bp deletion]), and *kmt2d*^*zy60*^ (Fig 1B [panels c, f, I; 2 bp deletion]). These mutant alleles are predicted to result in a premature stop codon leading to truncation in one of the PHD domain located at the N terminus of the protein. Loss of full-length Kmt2d protein was confirmed by immunohistochemistry (S1A Fig and S1B Fig).

In order to assess the phenotypes of *kmt2d* mutations and to determine whether any identified phenotypic traits followed expected mendelian ratios, we performed 3 different heterozygous by heterozygous crosses for each of 3 mutant alleles and screened for phenotypes, with an emphasis on organs and systems that are affected in KS patients. Interestingly, embryonic development was grossly normal until 2 days post fertilization (dpf). Starting at 3 dpf, embryos showed signs of cardiac edema (Fig 1B, arrow), microcephaly (Fig 1B, arrowhead), and body axis defects (Fig 1B [panels g, h, i])—attributes similar to KS clinical manifestations. Whole body edema was evident at 3 to 4 dpf (S2A Fig, S2B Fig, S2C Fig), and these phenotypes continued to develop until approximately 7 dpf.

To assess whether these phenotypes segregated with the mutant alleles, we selected 16 embryos with wild-type phenotypes and 32 embryos with KS phenotypes at 3 dpf and processed them individually for DNA extraction and HRMA analysis. All embryos with KS phenotypes were homozygous for the *kmt2d* mutant alleles, whereas embryos that appeared phenotypically normal were homozygous *wild type* or heterozygous at the *kmt2d* locus. Moreover, the percentage of embryos from a het by het cross with an abnormal phenotype was approximately 25% in 3 different clutches for each of the 3 alleles assayed (Fig 1C). These results were verified by performing Chi-square and binomial distribution tests against an expected mendelian ratio of 75% *wild-type* and heterozygous embryos (no phenotype) and 25% mutant embryos (with phenotype; Fig 1C).

Humans with KS have wide variation in phenotypes [7,31]. Similarly, zebrafish *kmt2d* mutants had a range of variable expressivity of phenotypic traits. To assess whether the observed variable expressivity occurred for different parental crosses and mutant alleles, we defined 3 phenotypic categories as follow: class I, WT phenotype corresponding to homozygous *wild-type* and heterozygous siblings (Fig 1B [panels a, b, c]); class II, homozygous mutant embryos exhibiting heart edema and microcephaly (Fig 1B [panels d, e, f]); class III, homozygous mutant embryos exhibiting heart edema, microcephaly, and shorter body axis (Fig 1B [panels g, h, i]). Embryos from 3 different parental crosses for each of 3 mutant alleles were screened for each category. Our results showed that variable expressivity of the phenotypic traits is a consistent characteristic in zebrafish *kmt2d* homozygous mutants (Fig 1C).

Our protein expression data showed that Kmt2d was strongly and ubiquitously expressed during development (S1C Fig, S1D Fig, S1E Fig, and S1F Fig), consistent with phenotypes in multiple organ systems. In order to discover pathway perturbations that lead to KS phenotypes, we pursued the *kmt2d*-dependent alterations in transcriptional profiles that immediately precede the onset of the KS phenotypes, reasoning that transcriptome changes that are proximal to the earliest onset of phenotypes would be most informative and not confounded by downstream secondary effects of transcriptomes. RNA-sequencing (RNA-seq) analysis was performed on single 1 dpf embryos, prior to the appearance of a KS phenotype. The transcriptome was analyzed in 6 individual homozygous *kmt2d*^*zy59*^ mutants and 6 individual homozygous *wild-type* siblings (total *n =* 12). For gene differential expression analysis, we performed DESeq2 using Likelihood Ratio Test (LRT) [32] at a false discovery rate (FDR) of 5%. Our results showed that, at 1 dpf, 276 genes were differentially expressed in *kmt2d*^*zy59*^ mutants when compared with their *wild-type* siblings (Fig 1D, MA plot pink and colored dots). Text-mining analysis (further described in Methods) revealed that 72.1% of the top 50 genes are associated with neural and/or cardiovascular system, whereas the remaining genes have been associated with reproductive system, muscle, and pharyngeal arches development (Fig 1D, MA plot color-coded dots; S1 Table). Furthermore, gene set enrichment analysis (GSEA) revealed a small group of gene sets that were enriched in *kmt2d*^*zy59*^ mutants (S2 Table). Interestingly, all the gene sets with a 5% FDR were exclusively associated with structural extracellular matrix (ECM) glycoproteins (Fig 1E; S2 Table), suggesting essential changes in ECM composition or topography even before phenotype manifestation. In addition, a specific Notch downstream target gene, *her4*.*4*, appeared to be up-regulated in *kmt2d* mutants (Log2 fold change = 044, *p* = 0.039), consistent with our subsequent analysis of Notch signaling.

Our gross examination of *kmt2d* mutants at 3 dpf and transcriptome analysis supports the hypothesis of a relatively small group of Kmt2d-dependent genes affected early in development that could explain the molecular etiology of *kmt2d*^*zy59*^ phenotype observed at later stages. Together, these data introduce a zebrafish *kmt2d* null mutant model and demonstrate its phenotypic and molecular utility as a model for studying Kabuki Syndrome.

### *kmt2d*^*zy59*^ mutants exhibit anomalous palate development and middle ear structural defects

**Fig 2:**
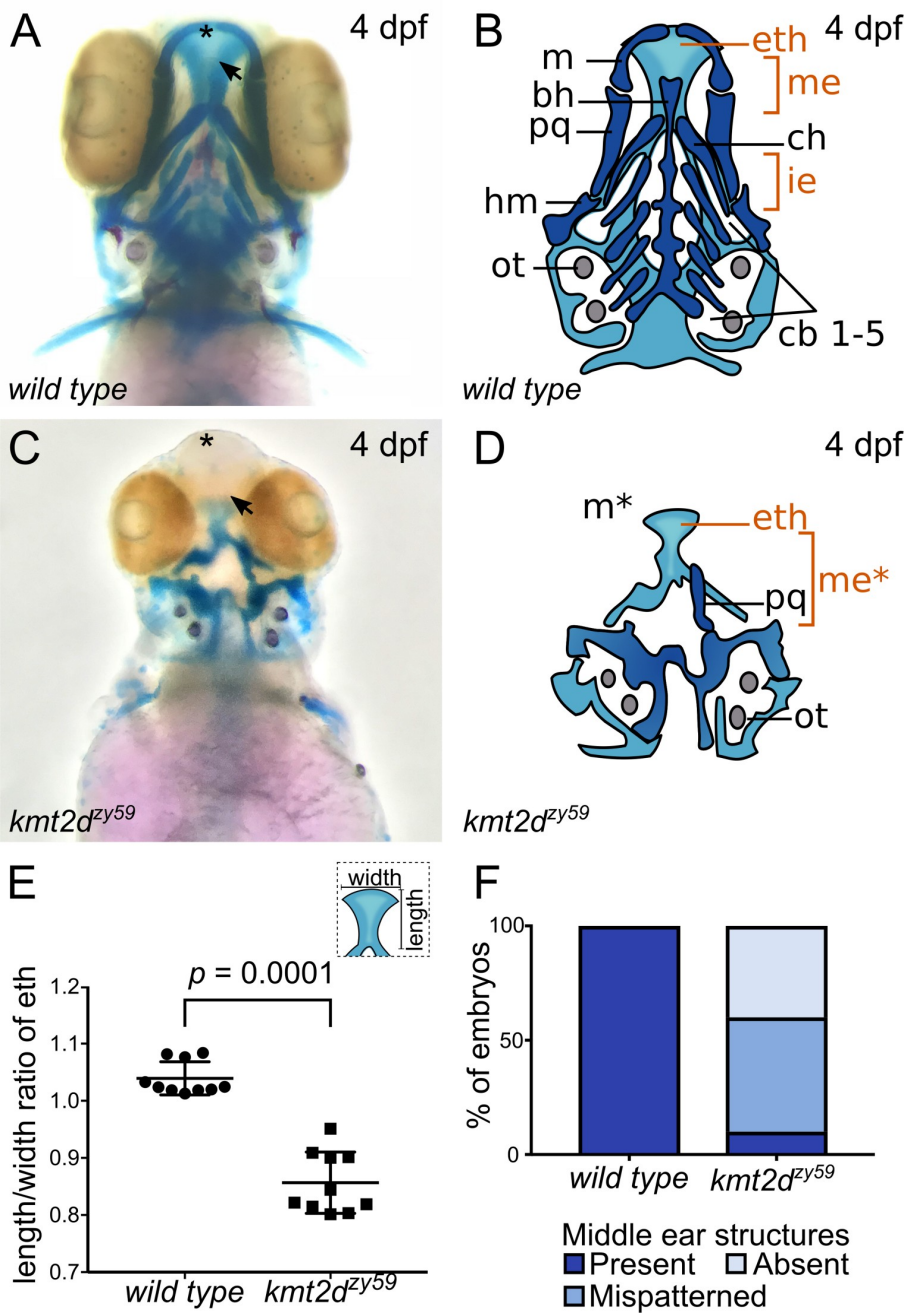
*kmt2d* mutants exhibit anomalous palate development and middle ear structures defects. (A-D) Alcian Blue/Alizarin Red staining of cartilage and bone showing zebrafish homologous structures for mammalian palate (neurocranium) and middle ear (jaw structures). Ventral view of zebrafish sibling control (A) and *kmt2d*^*zy59*^ mutant (C) embryos at 4 dpf with corresponding simplified cartoons (B, D). *kmt2d*^*zy59*^ mutants had severe hypoplasia of visceral cartilages (C; dark blue, D) and neurocranium (C; light blue, D) when compared with *wild-type* siblings (A, B). The ethmoid plate was present but displayed abnormal development (eth). The cartilages that pattern the jaw in the mandibular (m, pq) and hyoid (ch, hm) arches were absent (m*) or drastically reduced. Pharyngeal arches were absent (cb1-5). The specific structures that are considered mammalian homologs of palate and middle ear are highlighted in orange (eth, me). (E) Quantification of width/length ratio of the ethmoid plate in *wild-type* siblings and *kmt2d*^*zy59*^ mutants. In all mutant embryos analyzed, the ethmoid plate was present but had a significantly reduced length/width ratio when compared with *wild-type* siblings. Statistical analysis was carried out using two-tailed *t* test, *p* < 0.0001, *n =* 10 per genotype. Values for each data point can be found in S1 Data. (F) Embryos were classified according to the degree of development of homolog structures for mammalian middle ear. The categories were present, mispatterned, or absent. Qualitative assessment was plotted for percentage of embryos per genotype (*n =* 10 per genotype). Values for each data point can be found in S1 Data. bh, basihyal; cb, ceratobranchial; ch, ceratohial; dpf, days post fertilization; eth: ethmoid plate; hm, hyomandibula; ie, inner ear; m, Meckel’s; me, middle ear mammalian homologs; ot, otolith; pq, palatoquadrate.

Our morphological and transcriptome analyses suggested that abnormal pharyngeal arch development might contribute to the spectrum of cranial defects observed in *kmt2d*^*zy59*^ embryos (Fig 1B and 1D). Additionally, palate and middle ear structures are derived from pharyngeal arches and are affected in KS patients, serving as diagnostic features for the syndrome [2,4–6]. Zebrafish anterior neurocranium and lower jaw structures are well established models for mammal palate and middle ear development, respectively [33]. To assess whether Kmt2d loss affects pharyngeal arch development, we analyzed craniofacial skeleton phenotypes by Alcian Blue/Red Alizarin staining in *kmt2d*^*zy59*^ mutants and *wild-type* siblings. Craniofacial cartilage architecture was strongly affected in *kmt2d*^*zy59*^ mutants (Fig 2) and in *kmt2d*^*zy58*^ and *kmt2d*^*zy60*^ mutants (S2D Fig, S2E Fig, S2F Fig).

The neurocranium (Fig 2B and 2D; light blue) was underdeveloped, and the ethmoid plate (henceforth palate) failed to grow distally (Fig 2A and 2C, arrow). For *wild-type* siblings, the average length/width ratio of the palate was 1.03 (Fig 2E; *n* = 10), whereas *kmt2d*^*zy59*^ mutants exhibited a significantly shorter and wider configuration with an average length/width ratio of 0.85 (Fig 2E; *n* = 10). These results demonstrate that Kmt2d is essential for normal palate development in zebrafish embryos. Comparably, visceral cartilages were significantly affected in *kmt2d*^*zy59*^ embryos (Fig 2B and 2D; dark blue). The pharyngeal arch–derived structures that contribute to the support of the delicate gill tissues (Fig 2B–2D; cb1-5) were completely absent in *kmt2d* mutants. The cartilage that patterns the lower jaw was absent (Fig 2C and 2D; Meckel’s cartilage, asterisk) or drastically reduced (Fig 2C and 2D; palatoquadrate, hyomandibula, ceratohyal). This represents mispatterning of the structures that are homologous to the mammalian middle ear (Fig 2D; marked as me* in orange). To evaluate reproducibility of this phenotype, we categorized embryos based on the degree of development of Meckel’s, palatoquadrate, and hyomandibula cartilages and calculated percentage of individuals in each category in *wild-type* siblings and *kmt2d*^*zy59*^ embryos (Fig 2F). Our results showed that 90% of *kmt2d*^*zy59*^ embryos had absent or mispatterned cartilages in the lower jaw. Together, these results demonstrate that Kmt2d is required for normal development of palate and lower jaw in zebrafish, consistent with clinical findings in KS patients.

### *kmt2d*^*zy59*^ mutants have hypoplastic hearts and occluded lumens because of aberrant endocardial cell morphology

**Fig 3:**
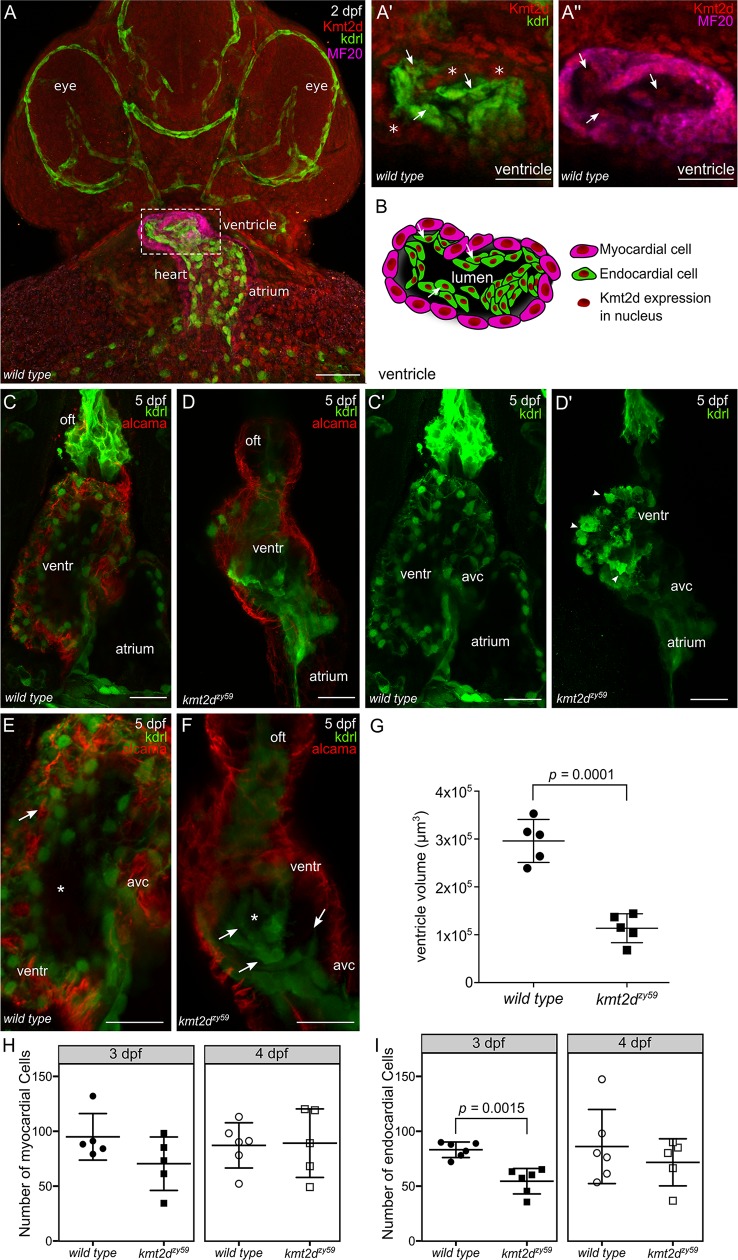
*kmt2d* mutants exhibit hypoplastic heart and aberrant endocardial cells morphology. (A) Confocal images of whole mount immunofluorescence for *wild-type* zebrafish Kmt2d protein expression at 2 dpf (ventral view). Kdrl (endothelium and endocardium) and MF20 (myosin) were use as context marker for cardiovascular tissues and myocardium. Kmt2d expression was found in the nuclei of myocardial (Aʹ, asterisk in example cells) and endocardial (A", arrows) cells in zebrafish heart (Aʹ and A" are zoomed images in ventricle area). Scale bar = 100 μm. (B) Cartoon showing Kmt2d nuclear expression in both myocardium and endocardium tissue of zebrafish heart. Arrows in Aʹ, A", and F are showing Kmt2d endocardial expression in the same set of cells. (C–D) Confocal images of wild-type *tg(kdrl*:*GFP)* sibling (C, Cʹ) and *kmt2d*^*zy59*^;*tg(kdrl*:*GFP)* (D, Dʹ) embryos at 5 dpf. Ventral view. *kmt2d*^*zy59*^ mutant had aberrant cardiac morphology with significantly reduced ventricle size (D). Myocardial cell labeled with Alcama antibody (C–D, red) showed normal cell morphology in both genotypes. Maximum intensity projections of GFP channel evidenced abnormal morphology of endocardial cells (Dʹ). Some endocardial cells exhibited cell protrusions (Dʹ, arrowheads). (E, F) High magnification images of the heart ventricle chamber in a middle-plane view from the three-dimentional data set in *wild-type* sibling embryo (E, higher magnification of C) and *kmt2d*^*zy59*^ mutant (F, higher magnification of D) at 5 dpf. Z-stack analysis of the data set revealed that endocardial cells from the ventricle are organized in concentric layers (F, arrows) filling up the cardiac lumen in *kmt2d*^*zy59*^;*tg(kdrl*:*GFP)* mutants (E, F, asterisk). (G) Ventricle cavity volume measurements in 5 embryos per genotype. Statistical analysis was carried out using two-tailed *t* test, *p* < 0.0001. Scale bar = 25 μm. Values for each data point can be found in S1 Data. (H, I) Ventricle myocardial (H) and endocardial (I) cells quantification at 3 and 4 dpf in zebrafish *wild-type* siblings and *kmt2d*^*zy59*^ mutants. Embryos were processed for immunofluorescence against myosine heavy chain (MF20) and GFP (Kdrl). Nuclei were stained with DAPI for cell quantification with Imaris software. (H) Myocardial cells, *t* tests per time point, *p*-values as follow: 3 dpf *p* = 0.129, effect size = 24.6. 4 dpf *p* = 0.906, effect size = −2. Bonferroni corrected *p*-values for 2 *t* tests *p* adjusted values, 3 dpf = 0.258 and 4 dpf = 1.000. (I) Endocardial cells, *t* tests per time point, *p*-values as follow: 3 dpf *p-* = 0.0007, effect size = 29. 4 dpf *p* = 0.4139, effect size = 14.6. Bonferroni corrected *p*-values for 2 *t* tests *p* adjusted values, 3 dpf = 0.0015 (value reported in figure) and 4 dpf = 0.8278. Values for each data point can be found in S1 Data. avc, atrio-ventricular canal; dpf, days post fertilization; kdrl, kinase insert domain receptor like; kmt2d, Histone-lysine N-methyltransderase 2D; oft, outflow tract; ventr, ventricle.

Congenital heart defects (CHD) is diagnosed in 28% to 80% of Kabuki Syndrome patients [34]. The spectrum of CHD is wide, with prevalence of aortic coarctation, hypoplastic left heart, and other left-sided obstructive defects [14,15,34]. Previous studies of a mouse cardiomyocyte conditional *kmt2d* deletion demonstrated that Kmt2d is required in cardiac precursors and cardiomyocytes [22]. Additionally, zebrafish *kmt2d* morphants were reported to have heart looping defects and abnormal development of atrium and/or ventricle [35]. Despite the growing body of evidence that Kmt2d functions during myocardium development, it has not yet been possible in mice to assess the effects of Kmt2d loss in all cardiac tissues in a null mutant context. To investigate the effects of Kmt2d loss during heart development, we first asked whether Kmt2d protein was expressed in both myocardial and endocardial tissues in zebrafish hearts. Whole mount immunofluorescence in *wild-type* zebrafish embryos revealed that Kmt2d was ubiquitously expressed in the nucleus of both myocardium and endocardium at 2 dpf (Fig 3A and 3B). In our *kmt2d* mutants, the cardiac edema that appears at 3 dpf could be the result of accumulated defects in heart morphogenesis or secondary effects from other altered embryological processes. To address whether heart edema could be the result of impaired cardiac function, the heart beat was recorded at 1, 2, 3, and 4 dpf in *kmt2d*^*zy59*^ embryos and *wild-type* siblings. We found statistically significant bradycardia in *kmt2d*^*zy59*^ embryos compared with their *wild-type* siblings (S3C Fig; ANOVA significant *p* = 0.000264, F (1,76) = 14.647). Interestingly, the mean difference between wild type and mutants was constant throughout this developmental period (ANOVA, interaction effect *p* = 0.746), suggesting bradycardia was not due to later-onset secondary effects but is a constitutive phenotype of *kmt2d*^*zy59*^ embryos.

Next, we analyzed myocardium and endocardium patterning using *tg(kdrl*:*GFP) crossed into the kmt2d*^*zy59*^ line to mark endocardium and endothelium. Cardiac morphology was significantly altered in *kmt2d*^*zy59*^ at 5 dpf, with overall heart size reduced and a hypoplastic ventricle (Fig 3C and 3D). To assess whether the hypoplastic ventricle was due to abnormal myocardium development, we analyzed ventricle cardiomyocyte cell numbers at 3 and 4 dpf in *wild-type* siblings and *kmt2d*^*zy59*^ mutants. Additionally, myocardial cell architecture was evaluated with the marker Alcama at 3 dpf. The numbers of ventricle myocardial cells in *wild-type* siblings and *kmt2d*^*zy59*^ mutants were equivalent (Fig 3H; *t* tests per time point, *p*-values as follow: 3 dpf *p* = 0.129, effect size = 24.6. 4 dpf *p* = 0.906, effect size = −2. Bonferroni corrected p-values for 2 *t* tests *p* adjusted values, 3 dpf = 0.258 and 4 dpf = 1.000.), suggesting that the hypoplastic ventricle phenotype in zebrafish *kmt2d*^*zy59*^ is not due to a reduction in the myocardial cell population.

At 3 dpf, zebrafish heart ventricle undergoes the tightly regulated process of trabeculation [36,37], whereby myocardial cells extrude and expand into the lumen of the ventricle [38] forming a network of luminal projections consisting of myocardial cells lined by endocardial cells [38,39]. After this process, myocardial cells of the outer ventricle curvature have a characteristic elongated shape [40]. In order to assess whether cardiomyocyte cell shape was affected in *kmt2d*^*zy59*^ mutants, we measured outer curvature ventricle myocardial cell area and sphericity in *wild-type* siblings and *kmt2d*^*zy59*^ at 3 dpf as previously described by Auman and colleagues [40]. Our results showed that ventricle myocardial cells morphology was equivalent in *wild-type* siblings and *kmt2d*^*zy59*^ mutants at 3 dpf (S3A Fig, *t* test *p =* 0.583 not significant [n.s.] t = 0.59 dF = 5 for area; S3D Fig *t* test, *p* = 0.946 n.s.t = 0.71, dF = 6 for myocardial cells sphericity). Overall these results suggest that the hypoplastic heart ventricle in *kmt2d*^*zy59*^ is not driven by a reduction in size, shape, or absolute numbers of myocardial cells.

In contrast to what we observed in myocardium, the endocardium of *kmt2d* mutant hearts displayed strikingly abnormal morphology (Fig 3Cʹ and 3Dʹ), with endocardial cells in the ventricle forming discrete aggregates in which some individual cells show protruding borders resembling mesenchyme-like morphology (Fig 3Dʹ; arrowheads). In *kmt2d*^*zy59*^ embryos, endocardial cells lose their tight interaction with the adjacent myocardium and form concentric layers (Fig 3E and 3F; arrows) that ultimately occlude the lumen of the ventricle (Fig 3E and 3F; asterisk). Thus, endocardium mispatterning led to lumen volume reduction in all analyzed mutant samples (Fig 3G; *t* test *p* = 0.0001, t = 7.54, dF = 8). Interestingly, quantification of ventricle endocardial cells in *wild-type* versus *kmt2d*^*zy59*^ mutants at 3 dpf showed a significant reduction in the number of EC cells in *kmt2d* mutants (Fig 3I, *t* tests per time point, *p*-values as follow: 3 dpf *p-* = 0.0007, effect size = 29. 4 dpf *p* = 0.4139, effect size = 14.6. Bonferroni corrected *p*-values for 2 *t* tests *p* adjusted values, 3 dpf = 0.0015 and 4 dpf = 0.8278). However, this reduction in endocardial cell number did not appear to be due to cell death, because anti active caspase3 IF did not find significant differences in the number of apoptotic cells in *kmt2d* mutant hearts in the ventricle (S3B Fig; arrowhead). Additionally, the observed difference in endocardial cell number at 3 dpf was not sustained at 4 dpf (Fig 3I, 4 dpf, not significant; 4 dpf *p* = 0.4139, effect size = 14.6. Bonferroni corrected *p*-values for 2 *t* tests *p* adjusted values, 3 dpf = 0.0015 and 4 dpf = 0.8278). This result indicates that the overall endocardium phenotype is due to (a) slower cell division rate and (b) an aberrant endocardial cell behavior that results in abnormal endocardial tissue patterning and occlusion of the ventricle lumen. This is consistent with the absence of higher rates of apoptosis (S3B Fig; arrowhead) and the observation that at 4 dpf the endocardial cell number was recovered. Altogether, these results indicate that Kmt2d is required for endocardium pattering during zebrafish cardiogenesis. Loss of *kmt2d* reduced overall heart size, resulting in hypoplastic ventricle with occluded cavity. These results suggest a previously unknown role of Kmt2d in endocardial patterning might contribute the cardiac phenotype observed in KS patients.

### *kmt2d*^*zy59*^ mutants are defective in vasculogenesis and angiogenesis

Although KS patients frequently manifest abnormalities of the aortic arches, including hypoplastic aortic arch or coarctation, a mechanistic understanding between *KMT2D* mutations and vascular anomalies is lacking. During early development, common progenitor cells (angioblasts) give rise to the cardiac endocardium and to the primary vascular endothelium through vasculogenesis [41–43]. After the primary vascular plexus is formed, a more complex vascular network is established through angiogenesis (production of vessels from preexisting ones) [44,45]. Considering their common developmental origin and our results demonstrating abnormal endocardium patterning, we assessed whether *kmt2d*^*zy59*^ mutants have normal vascular patterning. To do so, we analyzed general vasculature integrity through *o-dianisidine* staining [46] in wild-type siblings and *kmt2d*^*zy59*^ embryos at 6 dpf (S4A Fig, S4B Fig, S4C Fig, S4D Fig) and found vasculature mispatterning reflected by aggregates of red blood cells in the head and aortic arches (S4B Fig and S4D Fig; white arrowheads).

**Fig 4:**
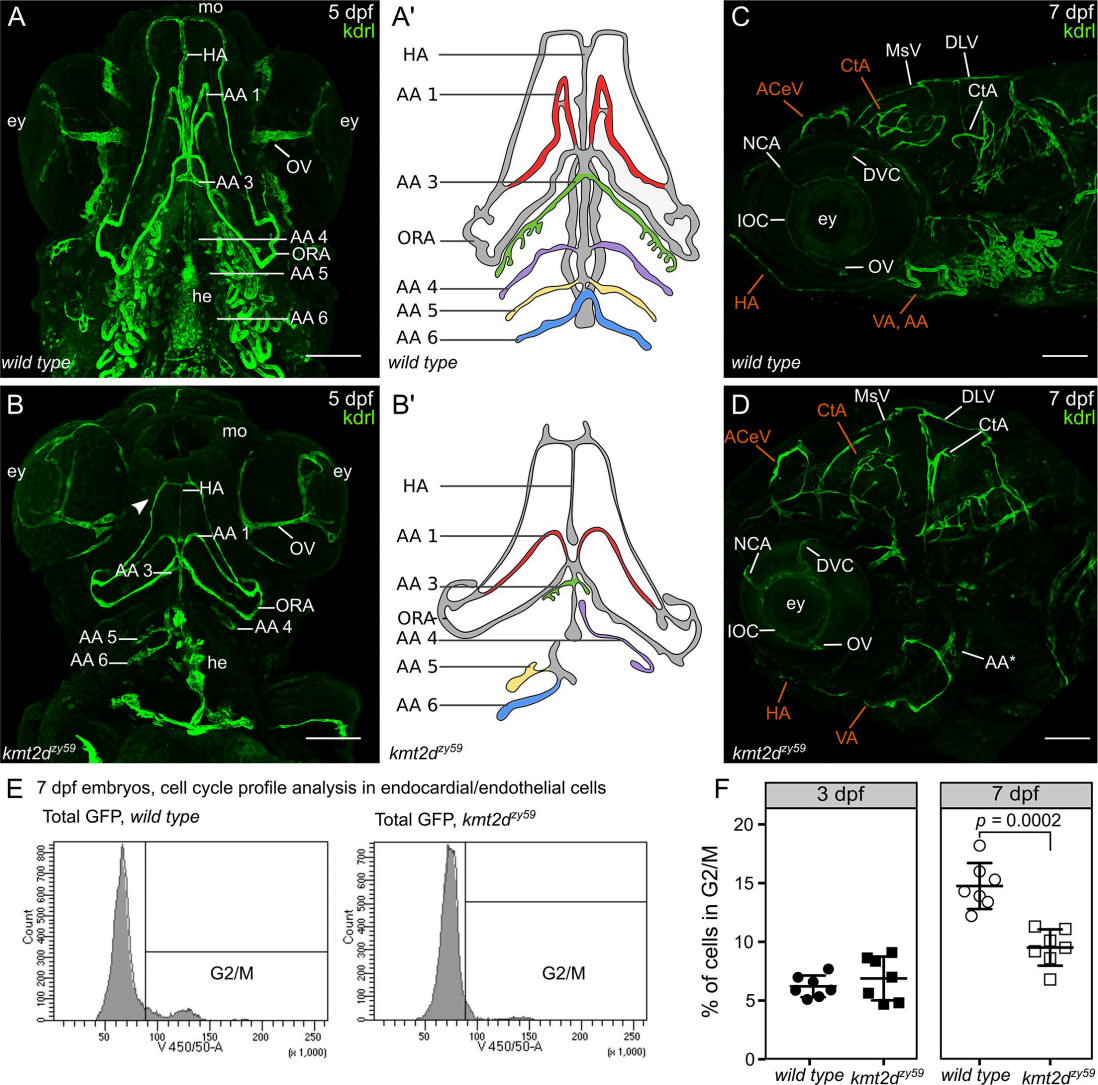
*kmt2d*^*zy59*^ mutants fail to develop AAs and exhibit misspatterned cephalic vasculature. (A-B) Ventral view of vasculature in *wild-type tg(kdrl*:*GFP)* sibling (A) and *kmt2d*^*zy59*^;*tg(kdrl*:*GFP)* mutants at 5 dpf. Cephalic vascular architecture in *kmt2d*^*zy59*^ embryos was abnormal with reduced elongation in the anteroposterior body axis, loss of bilateral symmetry, and reduced HA (A, B). In *kmt2d*^*zy59*^ mutants, AA1 (A, B) was shorter with minimum elongation towards the anterior area of the embryo, the AA3 (A, B) was rudimentary,whereas AA4−6 were reduced or absent (A, B). Mouth and eyes in *kmt2d*^*zy59*^ showed primitive characteristics with differences in the thickness of the OV in particular (A–B). Note the abnormal endocardial component of the heart (A, B; he). Aʹ and B are simplified cartoons of the main differences in the AAs development in *wild-type* (Aʹ) and mutant (Bʹ) backgrounds. Branchial vasculature loops were removed to allow better visualization of aortic arch points of origin. (C-D) Lateral view of cephalic vasculature in *wild-type tg(kdrl*:*GFP)* sibling and *kmt2d*^*zy59*^;*tg(kdrl*:*GFP)* mutants at 7 dpf. *kmt2d*^*zy59*^ exhibited a complete absence of the vascular loops associated with AAs 3–6 with only a rudiment of the AA3 present (D, AA*). The general cranial vascular network was mispatterned with a particular strong impact in the CtA, ACeV, and VA (D; in orange). In *kmt2d*^*zy59*^ mutant, most vessels had reduced lumens with the exception of the NCA (C, D), DVC (C, F) and IOC (C, F) that show thicker vessel diameter. Note the reduced HA from a lateral view (D, HA in orange; scale bars = 100 μm). (E) Cell cycle profile analysis by FACS for 7 individual embryos per genotype at 7 dpf. The gates set up for nuclear staining (DAPI) in kdrl positive cells (endothelial and endocardial cells) are shown. (F) Cell cycle profiles for Kdrl positive cells in *kmt2d*^*zy59*^ mutants showed no significant difference in the percentage of G2/M cells at 3 dpf. In contrast, at 7 dpf, there was a significant decrease in number of dividing cells in *kmt2d*^*zy59*^ mutants when compared with *wild-type* siblings. Unpaired two-tailed *t* test, *p* = 0.407 n.s. for 3 dpf; *p* = 0.0002 for 7 dpf, *n =* 7 per genotype. Values for each data point can be found in S1 Data. AA, aortic arch; AA1, mandibular arch; AA2, hyoid arch; AA3, first branchial arch; AA4, second branchial arch; AA5 third branchial arch; AA6, fourth branchial arch; ACeV, anterior cerebral vein; CtA, central artery; DCV, dorsal ciliary vein; DLV, dorsal longitudinal vein; dpf, days post fertilization; ey, eye; FACS, fluorescent activated cell sorting; GFP, green fluorescent protein; HA, hypobranchial artery; kdrl, kinase insert domain receptor like; IOC, inner optic circle; MsV, mesencephalic vein; NCA, nasal ciliary artery; ORA, opercular artery; OV, optic vein; VA, ventral aorta.

To assess whether the vascular phenotype in *kmt2d*^*zy59*^ mutants is driven by defects in vasculogenesis or angiogenesis, we analyzed vascular architecture in *kmt2d*^*zy59*^;*tg(kdrl*:*GFP)* mutants and *tg(Kdrl*:*GFP) wild-type* siblings by focusing on vasculogenesis of aortic arches (AAs) and angiogenesis of the cranial vessels network at 3, 4, 5, and 7 dpf. AA patterning, thickness, and development of general vasculature was dramatically altered in *kmt2d*^*zy59*^ mutants (Fig 4A and 4D; S4F Fig and S4H Fig) when compared with their *wild-type* siblings (Fig 4A and 4C; S4E Fig, S4F Fig and S4G Fig). Additionally, *kmt2d* mutants exhibited a primitive mouth with underdeveloped vasculature that failed to elongate anteriorly (Fig 4B, arrowhead). AAs were reduced or completely absent in *kmt2d* mutants. Consequently, all the vessels that branch into the gills were missing (Fig 4B; vessels on both sides of the heart). Three-dimensional volume rendering of confocal imaging confirmed that AA1 was fully formed but in a primitive state, whereas AAs 3, 4, 5, and 6 were atrophic on one side of the left-right symmetry axis and missing on its specular side (Fig 4Aʹ and 4Bʹ and S1 and S2 Videos). This result indicates that while the formation of the vascular plexus of the AAs was initiated, the overall vasculogenesis process is markedly abnormal in *kmt2d*^*zy59*^ mutants.

The vascular plexus of the brain in *kmt2d*^*zy59*^ mutants had all the major vessels present but with aberrant patterning. Defects were particularly evident in the central artery (CtA), anterior cephalic vein (ACeV), hypobranchial artery (HA) and ventral artery (VA; Fig 4C and 4D; orange segments indicate altered patterning). These observations suggest that even when blood vessels sprout and form, they fail to establish a normal vascular patterning (S4E Fig, S4F Fig, S4G Fig and S4H Fig).

To address whether the *kmt2d*^*zy59*^ vascular defects were driven by abnormal endothelial cell proliferation, we profile endothelial cell cycle by fluorescent activated cell sorting (FACS). In order to assess this, single embryos with distinguishable wild-type or mutant phenotypes were dissociated for FACS, using *kmt2d*^*zy59*^;*tg(kdrl*:*GFP)* embryos and *tg(Kdrl*:*GFP) wild-type* siblings at 3 dpf and 7 dpf (Fig 4E and 4F; S4 Fig). Strikingly, our results showed no significant difference in endothelial cell cycle profile at 3 dpf (unpaired *t* test, *p* = 0.4, t = 0.86 dF = 12 n.s.). In contrast, at 7 dpf, *kmt2d*^*zy59*^;*tg(kdrl*:*GFP)* embryos showed a significant reduction in proliferative endothelial cells (Fig 4F; *t* test, *p* = 0.0001, t = 5.56 dF = 12; *n =* 7 per genotype). Endothelial/endocardial cell proliferation was also analyzed by confocal analysis of phospho S10 Histone 3 (pH3) marker at 3dpf and 5dpf (S8 Fig and S7A Fig and S7C Fig). At 3 dpf, *kmt2d*^*zy59*^ mutants did not show statistically significance alterations in the number of endothelial/endocardial mitotic cells (S8 Fig, *t* test *p* = 0.076, dF = 7.92, *N* = 5 embryos per genotype). However, at 5 dpf, the difference in mitotic endothelial and endocardial cells between *wild-type* siblings and *kmt2d*^*zy59*^ mutants was statistically significance (Fig 7G and 7H, p = 0.001 in AA and p = 0.0307 in endocardium and S7A Fig and S7C Fig). These data indicate that the vascular misspatterning in *kmt2d*^*zy59*^ mutants is not driven by reduced cell proliferation at early developmental stages when the cardiovascular system is being established but by a reduction in proliferation at later stages. In contrast to normal proliferation at 3 dpf, there was a strong reduction in EC proliferation at 5 dpf and 7 dpf, particularly evident in the area of the vasculature that irrigates the gills (S7A Fig; white dashed line), which are normally established from 5 dpf to 7 dpf through active cell proliferation from vessels derived from AA3 to AA6 [47]. Overall, these results demonstrate key roles of Kmt2d in zebrafish for vascular patterning, with mutants displaying defects in both vasculogenesis and angiogenesis.

### *kmt2d*^*zy59*^ mutants fail to initiate AA3 to AA6 formation

In zebrafish, AA1 forms at 24 hpf, coincident with the time when circulation begins. A vestigial AA2 also forms at this time point, which is immediately replaced by the opercular artery (ORA). AA3 to AA6 emerge later, between 2 dpf and 2.5 dpf [47,48]. As described above, *kmt2d*^*zy59*^ mutants are able to form AA1 and the ORA, but they fail to form the subsequent AAs. Additionally, our data indicate that endothelial cell proliferation is normal in *kmt2d*^*zy59*^ mutants at 3 dpf, the time point at which the gross embryological phenotypes begin to appear. We explored 2 possible mechanisms for the absence of AA3 through AA6 in *kmt2d*^zy59^ mutants: (1) arch patterning fails to initiate at 2 dpf, or (2) alternatively, AA3 to AA6 vascular patterniing is initiated normally but at a later stage the arches become atrophic and regress. To test these possibilities, we performed live time-lapse analysis of AA development in *wild-type tg(kdrl*:*GFP)* siblings and *kmt2d*^*zy59*^;*tg(kdrl*:*GFP)* mutants from 2 dpf to 3 dpf.

**Fig 5:**
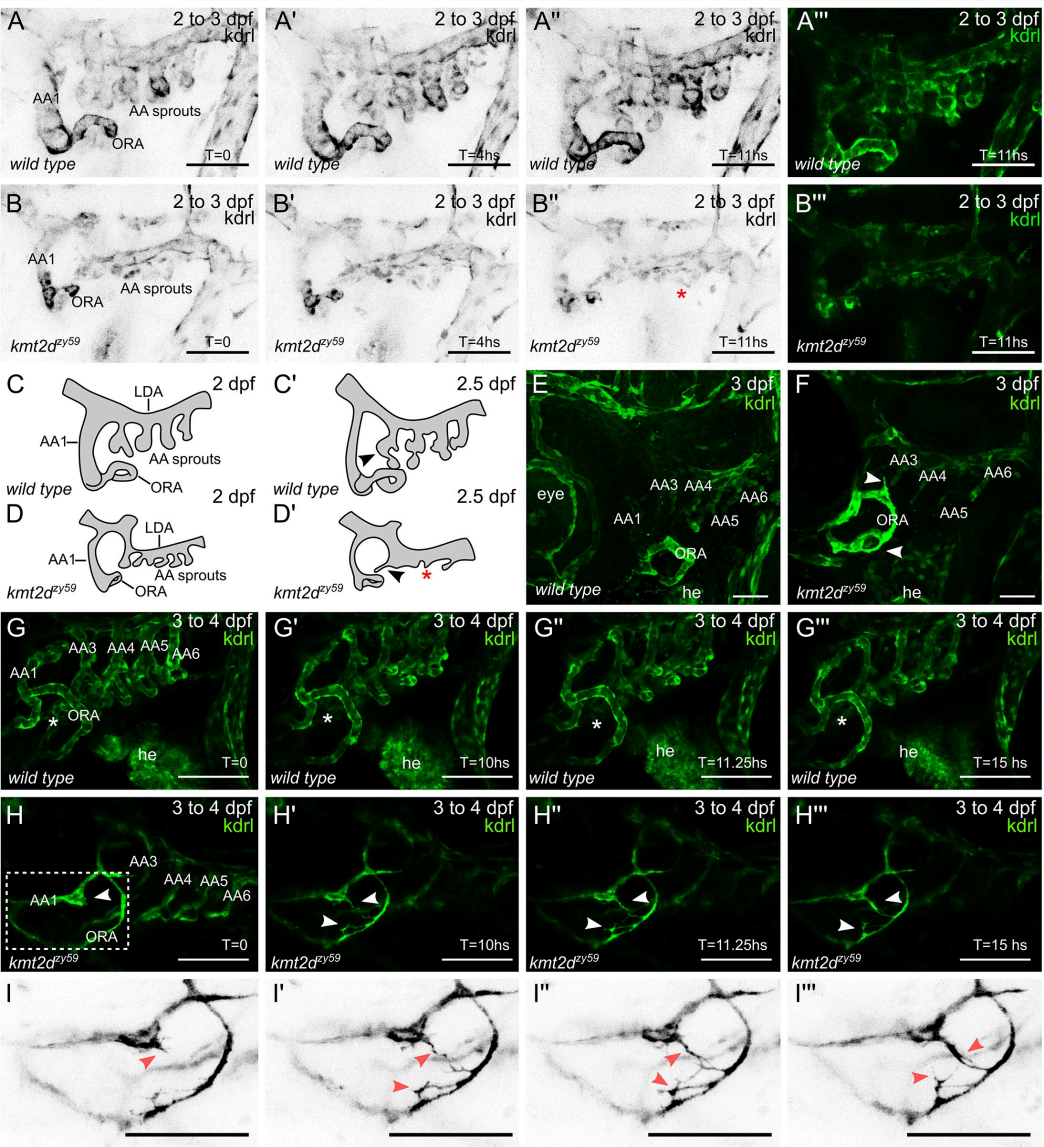
*kmt2d*^*zy59*^ mutants fail to develop AA3 to AA6. (A–B) Still images (MIP) from time-lapse live imaging performed from 2 dpf to 3 dpf. Cranial-lateral view at the level of AA development from *wild-type* sibling (A–A‴, S3 Video) and *kmt2d*^*zy59*^ mutant (B–B‴, S4 Video). Images were selected at the 0, 4, and 11 hour time points. Images were converted to grayscale and inverted for better visualization (A–A", B–B"). A‴ and B‴ show A" and B" without grayscale processing. Red asterisk (B") denotes abnormal vascular development of AA sprouts at the level of the ventral border of LDA. (scale bars = 50 μm). (C-D) Schematic cartoons highlighting the development of AA in *wild-type* embryos (C, Cʹ) and *kmt2d*^*zy59*^ mutants (D, Dʹ). C corresponds to A, Cʹ correspond to A", D corresponds to B and Dʹ to B". Red asterisk indicates ectopic AA sprouting. (E–F) Cranial-lateral view of vasculature in *wild-type tg(kdrl*:*GFP)* sibling (A) and *kmt2d*^*zy59*^;*tg(kdrl*:*GFP)* mutants at 3 dpf. After time-lapse experiment (A, B), embryos were released from agarose and processed for IF and confocal imaging. Wild-type embryos showed correct patterning and secondary sprouting of AA3 to AA6 (E). At 3 dpf, *kmt2d*^*zy59*^ mutants had abnormal development of primary sprouts of AA3 to AA6, which are thinner and atrophic. In contrast, AA1 and ORA were thicker and exhibit abnormal morphogenesis and endothelial cell protrusions (F, white arrowhead; scale bars = 25 μm). (G-I) Still images (MIP) from time-lapse live imaging performed from 3 dpf to 4 dpf. Cranial-lateral view at the level of AA development from *wild-type* sibling and *kmt2d*^*zy59*^ mutant. Images were selected at the 0, 10, 11.25, and 15 hour time points. Asterisks (G–G‴) denote normal vascular development in the area between AA1 and ORA. No blood vessel forms in this area in *wild-type* background. Arrowheads (H–H‴, I–I‴) indicate endothelial cells extending multiple filopodia and forming a new and ectopic blood vessel. White dashed rectangle (H) specifies zoomed area in (I). Images were set on grayscale and the look-up table was inverted for better contrast of tip cell-like endothelial cells in *kmt2d*^*zy59*^ mutant (I–I‴; scale bars = 100 μm). AA, aortic arch; AA1, mandibular arch; AA2, hyoid arch; AA3, first branchial arch; AA4, second branchial arch; AA5, third branchial arch; AA6, fourth branchial arch; dpf, days post fertilization; he, heart; hpf, hours post fertilization; kdrl, kinase insert domain receptor like; LDA, lateral ventral aorta; MIP, maximum intensity projection; ORA, opercular artery.

At 2 dpf, *kmt2d*^*zy59*^ mutants have normal gross morphology, but time-lapse imaging revealed thinner blood vessels and abnormal AA sprouting when compared with their wild-type siblings (Fig 5A–5D). Interestingly, *kmt2d*^*zy59*^ mutants were not able to form the primary AA sprouts during the duration of data acquisition (Fig 5B–5B‴, 5D and 5Dʹ, S4 Video), whereas *wild-type* embryos succeeded in developing and extending AA3 through AA6 (Fig 5A–5A‴, 5C and 5Cʹ, S3 Video). This result suggests that *kmt2d* mutants do not initiate the formation AA3 to AA6 within the same time interval as their wild-type counterpart. On the other hand, the time-lapse videos showed unusually high levels of endothelial cell activity in the area were the AAs should emerge (ventral boundary of the lateral dorsal aorta [LDA]), suggesting that AA could develop at a later time point. To investigate this possibility, embryos subjected to time-lapse analysis were recovered for 5 hours and processed for IF and confocal analysis. At approximately 3 dpf, *kmt2d* mutants showed primary sprouting of abnormal AA3 to AA6 that were not present at earlier stages (Fig 5E and 5F). This indicates that *kmt2d* mutants eventually form atretic AAs around 3 dpf that will ultimately become vestigial at later time points (Fig 4A and 4B).

### Ectopic endothelial sprouting in *kmt2d*^*zy59*^ mutants

Our superresolution confocal analysis of the 3 dpf embryos found distinctive aberrant sprouting of endothelial cells from AA1 and ORA in *kmt2d*^*zy59*^ embryos (Fig 5F, white arrowheads). This observation led us to test the hypothesis that angiogenesis defects in in *kmt2d* mutants involve ectopic blood vessels formation. To test this hypothesis, we performed time-lapse analysis of AA development in *wild-type tg(kdrl*:*GFP)* siblings and *kmt2d*^*zy59*^;*tg(kdrl*:*GFP)* mutants at a later time point, from 3 dpf to 4 dpf. At 3 dpf (72 hpf), aberrant blood vessel phenotypes were apparent in *kmt2d* mutants, with both decreased vessel lumens and abnormal patterning (Fig 5G and 5H). Strikingly, by 10 hours later (82 hpf), *kmt2d*^*zy59*^;*tg(kdrl*:*GFP)* mutants showed ectopic endothelial cell sprouting, emerging from the AA1 and ORA (Fig 5G, 5G", 5H' and 5H"; arrowheads; Fig 5I and 5I", red arrowheads). These cells possessed many long and dynamic filopodia extending towards apposing cells with similar comportment. This aberrant hyperactive sprouting resembles tip cell morphology seen during normal angiogenesis [49]. However, in the context of *kmt2d*^*zy59*^ mutants, the observed tip cell-like behavior occurred in aberrant (ectopic) positions, ultimately producing ectopic blood vessels (Fig 5H‴ and 5I‴, S5 Video, wild-type control; S6 Video *kmt2d* mutant). This ectopic and abnormal endothelial cell behavior was also observed at the level of AA3 as well as in other regions of the recorded area (S6 Video *kmt2d* mutant), suggesting that the tip-like phenotype can potentially occur in any endothelial cell of *kmt2d*^*zy59*^ mutants.

Ectopic blood vessel formation is a well described response to hypoxia conditions [50]. Considering the reduced lumen size in *kmt2d* mutant blood vessels, we asked whether ectopic blood vessel formation was a consequence of hypoxia response mechanisms. To test this, we induced hypoxia by treatment with dimethyloxalylglycineinduced (DMOG), an inhibitor of HIF-prolyl hydroxylases, which thereby stabilizes Hif-1 and triggers hypoxia response even in normoxia conditions [51]. *Tg(kdrl*:*GFP)* wild-type siblings and *kmt2d*^*zy59*^;*tg(kdrl*:*GFP)* mutant embryos were treated from 3 dpf to 4 dpf with DMOG and DMSO as control. After treatment, embryos were washed and processed for IF and confocal imaging. At 4 dpf, *wild-type* embryos treated with DMOG showed ectopic vessel sprouting at the level of the optic vein (OV; S5A Fig and S5B Fig, white arrow). In contrast, AA1 and ORA were not altered by DMOG treatment (S5A Fig and S5B Fig), suggesting that these vessels have a higher resistance threshold to hypoxia. Interestingly, *kmt2d* mutant embryos treated with DMOG did not show any additional vessel sprouting (S5B Fig and S5D Fig) beyond the ectopic blood vessel sprouting from the AA1 and ORA in normoxia (S5B Fig and S5D Fig; white arrow), indicating that hypoxia does not increase or alter the ectopic blood vessels formed in *kmt2d* mutants. These results suggest that the aberrant endothelial cell behavior and ectopic blood vessel sprouting seen in *kmt2d* mutants are not due to hypoxia response and that Kmt2d functions to suppress ectopic or hyperactive angiogenesis. Altogether, these results demonstrate that Kmt2d is required for the timely and normal development of AA3 to AA6 in zebrafish.

### Notch signaling is hyperactivated in endocardial and endothelial cells of *kmt2d* mutants

**Fig 6:**
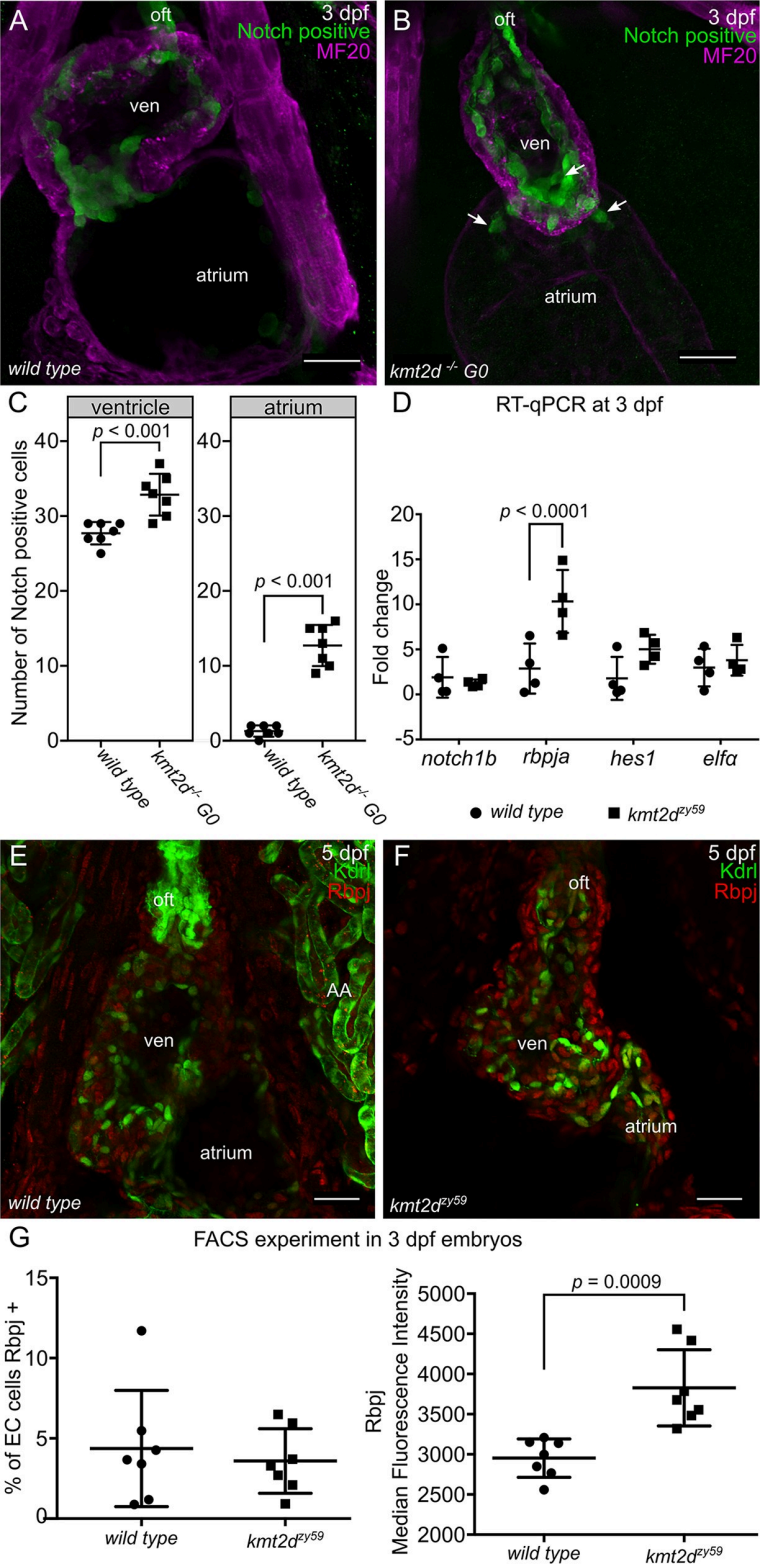
Notch signaling is hyperactive in *kmt2d* mutant endothelium/endocardium. (A-B) Confocal images of the heart of *wild-type* noninjected control *tg(tp1*:*EGFP)*^*um14*^ embryo (A) and F0 *kmt2d* mosaic mutants *tg(tp1*:*EGFP)*^*um14*^ embryo injected with CRISPR/Cas9 targeting *kmt2d* (B). F0 *kmt2d* mosaic mutants injected embryos showed hypoplastic heart as seen in *kmt2d*^*zy59*^ null mutants (B). Notch positive cells (green) were mostly distributed in the atrio-ventricular valve of a 3 dpf embryonic heart. Some endocardial cells in the ventricle and outflow tract were also observed (A). In F0 *kmt2d* mosaic mutants, hearts showed a significant increase in the number of Notch positive cells in both ventricle and atrium. Ventral view of the heart, only middle sections of the whole data set are shown. Scale bar = 25 μm. (C) Quantification of the amount of Notch positive cells in ventricle and atrium of control and injected embryos. *N =* 7 per group, unpaired two-tailed *t* test, *p* = 0.0001 in ventricle t = 4.95, dF = 36, atrium *p* = 0.0001, *t* = 10.99, dF = 36. Values for each data point can be found in S1 Data. (D) RT-qPCR analysis of *wild-type* sibling control embryos and *kmt2d*^*zy59*^ mutants for some components of the Notch signaling pathway. The Notch transcription factor *rbpja* was significantly up-regulated *kmt2d*^*zy59*^ embryos, corroborating the results obtained in the F0 *kmt2d* mosaic mutant analysis. There were no significant differences found for *notch1b* and *hes1*. *N =* 4 per genotype with 2 technical replicates per gene and per genotype assessed; *elfα* was use as control gene. Ct values were normalized using *α-tubulin* as gene of reference; fold change of relative expression was calculated using the ΔΔCt method. Multiple *t* test *p* < 0.0001 for *rbpja*, t = 6.04, dF = 24. Values for each data point can be found in S1 Data. (E–F) Confocal images of *wild-type* sibling control embryos (E) and *kmt2d*^*zy59*^ mutants (F) showing Rbpj protein expression levels and patterning at 5 dpf. Ventral view of the heart, only MIP of half data set is shown. Scale bar = 25 μm. (G) Summarized data and statistics from FACS experiment performed in 14 individual samples (7 *wild-type* siblings and 7 *kmt2d* mutants). *Wild-type tg(kdrl*:*GFP)* siblings (A) and *kmt2d*^*zy59*^;*tg(kdrl*:*GFP)* mutants were collected at 3 dpf, were processed for IF, were digested, and were prepared for FACS. Unpaired two-tailed *t* test, % of EC cells Rbpj positives *p* = 0.63, n.s. t = 0.49, dF = 12; Rbpj MFI *p* = 0,0009, t = 4.35, df = 12. Values for each data point can be found in S1 Data. Altogether our results show that Notch pathway is hyperactivated in endocardial cells of *kmt2d*^*zy59*^ mutants and demonstrate that this increased Notch activity is consequence of up-regulated Notch pathway transcription factor Rbpj specifically in EC cells. To our knowledge, these results provide the first evidence of a regulatory link between Kmt2d and Notch signaling during developmental processes in vertebrates. Ct, cycle threshold; ΔΔCt, delta-delta cycle thrshold; dpf, days post fertilization; EC, endocardial cells; F0, filial 0; FACS, fluorescent activated cell sorting; G0, generation 0; IF, immunofluorescense; MIP, maximum intensity projection; oft, out flow tract; RT-qPCR, reverse transcription-quantitative polymerase chain reaction; ven, ventricle.

The ectopic tip cell-like phenotype led us to investigate an iconic molecular mechanism involved in tip-stalk cell identity as well as in endocardium patterning during cardiogenesis: Notch signaling pathway. Multiple studies in mice, zebrafish, cell culture, and tumor models have shown that Notch pathway is a key regulator of vertebrate vasculogenesis and angiogenesis [52–58]. Considering our discovery of ectopic angiogenesis in *kmt2d* mutants and previous observations that Notch signaling regulates endocardial and endothelial cell growth, differentiation, and patterning, we investigated whether *kmt2d* mutants have altered Notch signaling activity. We injected *kmt2d* CRISPR/Cas9 into a Notch signaling reporter line *tg(tp1*:*EGFP)*^*um14*^ [59] and performed F0 *kmt2d* mosaic mutants analysis [60] (S6 Fig) to assess Notch signaling activity in *kmt2d* mutants. At 3 dpf, Notch signaling in the zebrafish heart was strongly active in endocardial cells of the outflow tract and AV canal, with weaker activity in some endocardial cells of the ventricle (Fig 6A; GFP positive cells). Interestingly, in *kmt2d* mutants, the number of endocardial cells with positive Notch activity was significantly increased in both ventricle and atrium (Fig 6B and 6C *t* test, *p* = 0.0001 in ventricle t = 4.95 dF = 36, atrium *p* = 0.0001, t = 10.99 dF = 36 *n =* 7 per treatment). These *trans*gene reporter results indicate that Notch signaling is cell autonomously hyperactivated in *kmt2d* mutant endocardial cells. To validate that endogenous Notch signaling is altered in *kmt2d* mutants, we assayed endogenous components of the Notch pathway in *kmt2d*^*zy59*^ mutants by reverse transcription-quantitative polymerase chain reaction (RT-qPCR) and IF at 3 dpf. Of these components, only transcripts of Notch-specific transcription factor *rbpja* were increased in *kmt2d*^*zy59*^ mutants, whereas the notch1b receptor and the downstream target *hes1* mRNA levels were comparable to the transcripts level found in *wild-type* siblings (Fig 6D). To investigate whether elevated expression of *rbpja* mRNA resulted in elevated Rbpj protein expression, we performed immunofluorescence against Rbpj Notch transcription factor and compared protein levels in the heart in *wild-type tg(Kdrl*:*GFP)* siblings and *kmt2d*^*zy59*^*;tg(Kdrl*:*GFP)* mutants. At 5 dpf, Rbpj protein was present in all heart tissues, with enhanced Rbpj protein accumulation in *kmt2d*^*zy59*^ mutants, with well-defined nuclear localization. To quantify this elevated expression in endothelial/endocardial cells and to determine whether this increased Rbpj protein expression started at earlier stages, we performed single embryo FACS of *tg(Kdrl*:*GFP) wild-type* siblings and *kmt2d*^*zy59*^*;tg(Kdrl*:*GFP)* mutants at 3dpf. Embryos were processed for immunofluorescence against GFP (driven by Kdrl in endothelial/endocardial cells) and Rbpj. After gating for single cells, samples were gated for GFP to exclusively study the endothelial/endocardial single cell population. Subsequently we measured in EC the percentage of Rbpj positive cells and the Rbpj Median Fluorescence Intensity (MFI) as an indicative of protein expression levels (Fig 6D). Consistent with our previous cell counting results, we observed that the overall number of Rbpj positive cells in EC from *wild-type* sibling and *kmt2d*^*zy59*^ mutants is equivalent at 3 dpf (Fig 5A, % of EC Rbpj positive, unpaired *t* test, *p* = 0.69, t = 0.42 dF = 6 n.s.). Crucially, the MFI values in *kmt2d*^*zy59*^ mutant ECs were significantly higher, indicating elevated Rbpj protein levels in Kmt2d-deficient endothelial/endocardial cells (Fig 6D, *t* test *p* = 0.0015, t = 5.51, dF = 6).

### Pharmacological inhibition of Notch signaling rescues cardiovascular development in Kabuki Syndrome zebrafish

**Fig 7:**
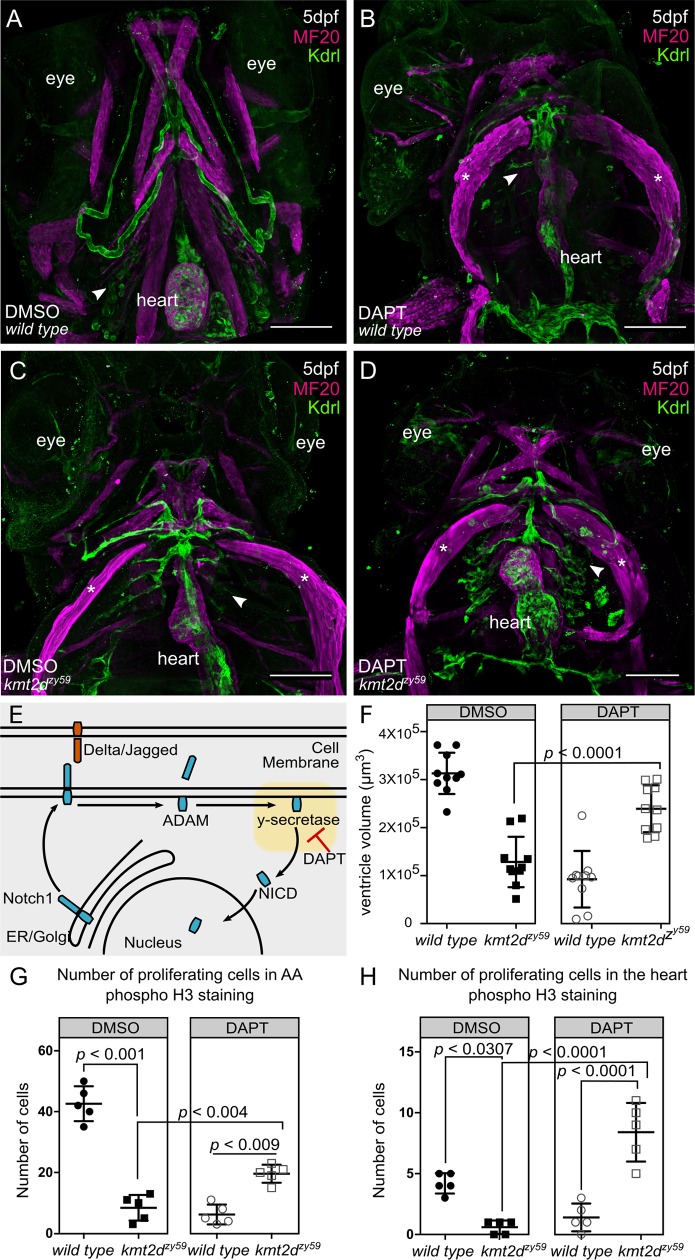
Inhibition of Notch pathway with DAPT rescues cardiovascular phenotypes in *kmt2d* mutants by enhancing cell proliferation in endothelial and endocardial cells. (A-D) Confocal images of *wild-type* sibling (A, B) and *kmt2d*^*zy59*^ mutant (C, D) embryos at 5 dpf. DMSO as solvent control (A, C) and DAPT for Notch signaling inhibition (B, D) were applied to embryos of indicated genotypes. DAPT treatment affected cardiovascular development in *wild-type* embryos (B) as a consequence of Notch signaling inhibition. *kmt2d*^*zy59*^ mutants that were treated with DAPT had rescued AA development and partially rescued heart development (D) when compared with the *kmt2d*^*zy59*^ DMSO control group (C). Arrowheads indicate normal (A), disrupted (B, C), and rescued (D) AA. Note that cardiovascular phenotype was rescued in DAPT-treated mutants despite cardiac edema, as evidenced by the sternohyoideus deformation (asterisk; B, C, D, MF20 in magenta). (E) Schematic of Notch signaling pathway showing DAPT inhibition of γ-secretase activity, the second cleavage step of Notch receptor processing. DAPT thus prevents NICD release to the cytoplasm for nuclear import. (F) Cardiac ventricle volume measurements for DMSO control groups (*wild type* and *kmt2d*^*zy59*^) and DAPT treatment groups (*wild type* and *kmt2d*^*zy59*^). The volume of the ventricle chamber was significantly rescued in *kmt2d*^*zy59*^ mutant embryos after Notch pathway inhibition with DAPT. Embryos were randomly selected from 3 different clutches; measurements were blind to embryo genotypes, which were assayed by HRMA after measurement. Two-way ANOVA multiple comparison test adjusted *p*-values per each condition as follow: *wild-type* DMSO versus wild-type DAPT, *p* < 0.0001; *wild-type* DMSO versus *kmt2d*^*zy59*^ DMSO, *p* < 0.0001; *wild-type* DMSO versus *kmt2d*^*zy59*^ DAPT, *p* = 0.0159; *wild-type* DAPT versus *kmt2d*^*zy59*^ DMSO, *p = 0*.*5482*; *wild-type* DAPT versus *kmt2d*^*zy59*^ DAPT, *p* < 0.0001; *kmt2d*^*zy59*^ DMSO versus *kmt2d*^*zy59*^ DAPT, *p* = 0.0001. Values for each data point can be found in S1 Data. (G) Number of endothelial cells proliferating in the AA region in each group treatment. There were significantly more proliferating endothelial cells in the AA region of *kmt2d*^*zy59*^ mutant after DAPT treatment, indicating that the phenotype was rescued by increasing cell proliferation. Two-way ANOVA multiple comparison test adjusted *p*-values per each condition as follow: *wild-type* DMSO versus wild-type DAPT, *p* = 0.0001; *wild-type* DMSO versus *kmt2d*^*zy59*^ DMSO, *p* = 0.0001; *wild-type* DMSO versus *kmt2d*^*zy59*^ DAPT, *p* = 0.0006; *wild-type* DAPT versus *kmt2d*^*zy59*^ DMSO, *p = 0*.*6043*; *wild-type* DAPT versus *kmt2d*^*zy59*^ DAPT, *p* = 0.0091; *kmt2d*^*zy59*^ DMSO versus *kmt2d*^*zy59*^ DAPT, *p* = 0.0047. Values for each data point can be found in S1 Data. (H) Number of endocardial cells proliferating in the heart per experimental group. There were significantly more proliferating endocardial cells in *kmt2d*^*zy59*^ mutant hearts after DAPT treatment, indicating that the phenotype was rescued by increasing endocardial cell proliferation. Two-way ANOVA multiple comparison test adjusted *p*-values per each condition as follow: *wild-type* DMSO versus wild-type DAPT, *p* = 0.0051; *wild-type* DMSO versus *kmt2d*^*zy59*^ DMSO, *p* = 0.0307; *wild-type* DMSO versus *kmt2d*^*zy59*^ DAPT, *p* = 0.0013; *wild-type* DAPT versus *kmt2d*^*zy59*^ DMSO, *p = 0*.*8106*; *wild-type* DAPT versus *kmt2d*^*zy59*^ DAPT, *p* < 0.0001; *kmt2d*^*zy59*^ DMSO versus *kmt2d*^*zy59*^ DAPT, *p* < 0.0001. Cell count was blind and embryos were randomly selected from 2 different clutches (E, F). Genotype was confirmed after measurement by HRMA. Values for each data point can be found in S1 Data. AA, aortal arch; ADAM, containing a disintegrin and metalloprotease; dpf, days post fertilization; ER, endoplasmic reticulum; HRMA, High Resolution Melt Analysis; kdrl, kinase insert domain receptor like; MF20, Myosin Heavy Chain Antibody; NICD, Notch intracellular domain.

Our results showed hyperactivation of Notch activity in endocardial and endothelial cells of *kmt2d* mutants, suggesting a molecular mechanism for the cardiovascular phenotype in KS. To test whether interference with this mechanism could rescue the cardiovascular phenotype in *kmt2d*^*zy59*^, we inhibited the Notch pathway at the level of the Notch receptor cleavage by blocking y-secretase activity with DAPT (Fig 7E) [58,61]. *kmt2d*^*zy59*^;*tg(kdrl*:*GFP)* embryos and *wild-type tg(kdrl*:*GFP)* siblings were treated with DAPT or DMSO (control group) from 1 dpf to 2 dpf and were washed and assessed at 5 dpf for cardiovascular development. *Wild-type* embryos treated with DAPT had other phenotypes predicted from inhibiting Notch as previously described by Arslanova and colleagues [61]: abnormal somite development, heart edema, disrupted vasculature. Confocal images of *wild-type* DAPT-treated embryos revealed aberrant heart morphology with hypoplastic ventricle and stretched cardiac tube, likely a consequence of pericardic edema (Fig 7A and 7B) evidenced by myosin staining of the sternohyoideus muscle (Fig 7A–7D asterisks, magenta labeling). Strikingly, DAPT treatment partially rescued AA development and heart morphology in *kmt2d*^*zy59*^ mutant embryos (Fig 7D). Heart ventricle volume was significantly increased in *kmt2d*^*zy59*^ DAPT-treated embryos compared with the control groups: DMSO *kmt2d*^*zy59*^ embryos and *wild-type* DAPT-treated embryos (Fig 7F; two-way ANOVA multiple comparison test, *n =* 10 per condition, adjusted *p*-values per group; see figure legend for *p-value* details).

We then asked whether the rescue of AA and heart development by Notch inhibition was due to a change in the proliferative capability of endothelial/endocardial cells. To test this, we analyzed cell proliferation by pH3 labeling and counted GFP positive cells (endothelial and endocardial cells) co-localizing with pH3 in a delineated area (S7A Fig; white dashed line). Our analysis showed that DAPT treatment of *kmt2d* mutants induces a significant increase in the number of proliferating endothelial and endocardial cells in the AA area and heart, respectively.

### Pharmacological inhibition of Notch signaling reestablishes Rbpj protein levels in *kmt2d* mutant endothelial and endocardial cells

Since *kmt2d*^*zy59*^ mutants showed increased levels of the Notch transcription factor Rbpj in endocardial and endothelial cells (Fig 6E–6G), we asked whether inhibition of Notch signaling with DAPT could rescue this phenotype. To test this, *kmt2d*^*zy59*^;*tg(kdrl*:*GFP)* embryos and *wild-type tg(kdrl*:*GFP)* siblings were treated with DAPT (or DMSO as a control) from 1 dpf to 2 dpf and were washed and grown until 3 dpf. Embryos were then fixed and genotyped by tail-clipping followed by DNA extraction and HRMA. Samples were processed for immunofluorescence against Rbpj and GFP (*Kdrl*:*GFP* transgenic enhancement) and analyzed by confocal imaging and FACS.

**Fig 8:**
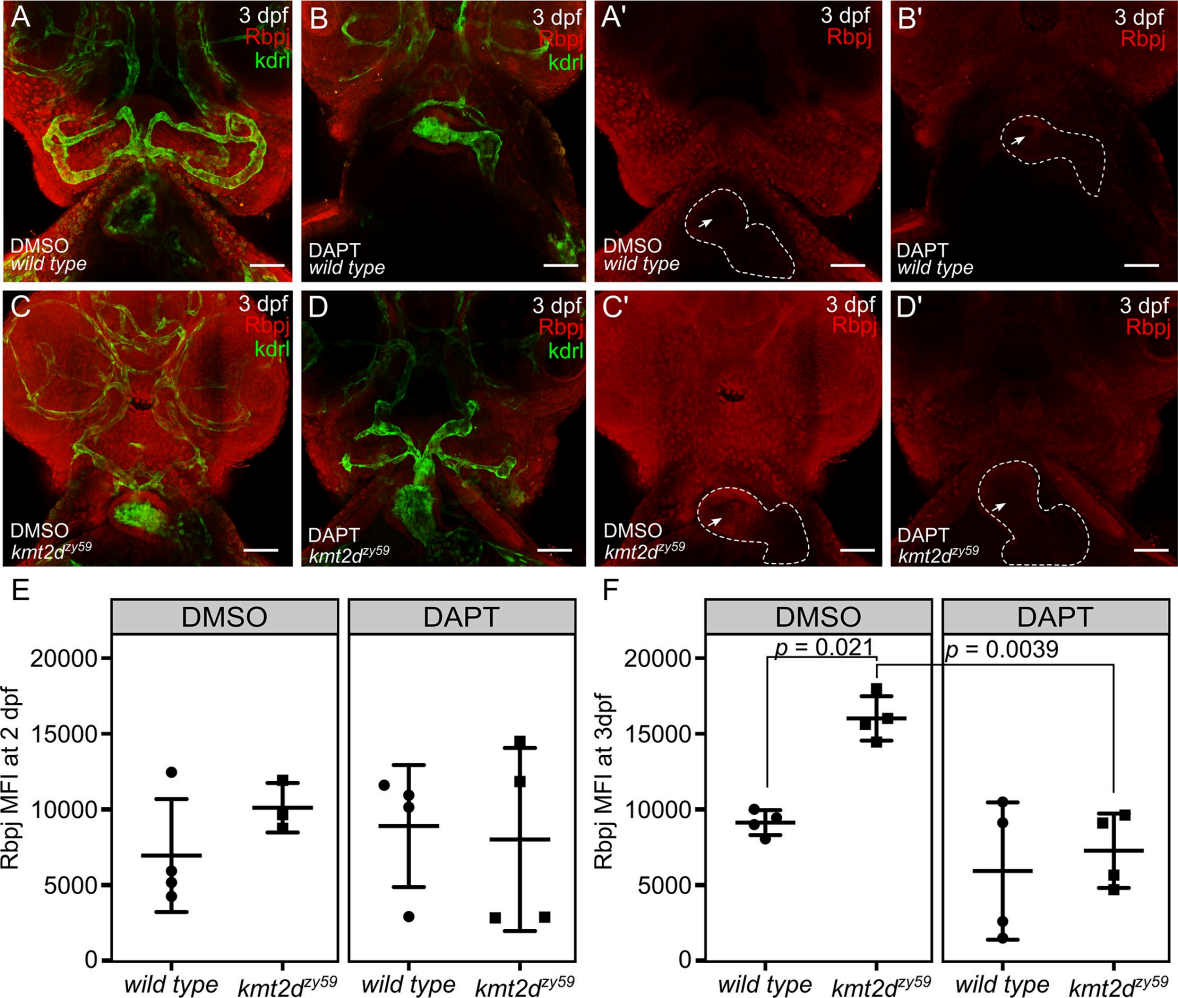
Pharmacological inhibition of Notch signaling reestablishes normal Rbpj protein levels in kmt2d mutant endothelial and endocardial cells. (A–D) Confocal images of *wild-type* sibling (A, B) and *kmt2d*^*zy59*^ mutant (C, D) embryos at 3 dpf. Cephalic ventral views. DMSO as solvent control (A, C) and DAPT for Notch signaling inhibition (B, D) were applied to embryos of indicated genotypes. Rbpj protein levels (Red channel) are higher in DMSO-*kmt2d*^*zy59*^ mutants (C, Cʹ). Arrow in Aʹ–Dʹ is indicating endocardial Rbpj expression—note higher endocardial Rbpj levels in Cʹ. DAPT treatment restored Rbpj signal levels in *kmt2d*^*zy59*^ embryos (D, Dʹ) as a consequence of Notch signaling inhibition. Dashed line in Aʹ–Dʹ indicates heart outline. Red, Rbpj; green, GFP (to enhance endocardial/endothelial Kdrl:GFP transgenic label). (E-F) Summarized data and statistics from FACS experiments performed in 16 individual samples per time point (4 DMSO *wild-type* siblings and 4 DMSO *kmt2d* mutants; 4 DAPT *wild-type* siblings and 4 DAPT *kmt2d* mutants per time point). *Wild-type tg(kdrl*:*GFP)* siblings (A) and *kmt2d*^*zy59*^;*tg(kdrl*:*GFP)* mutants from each treatment (DMSO or DAPT) were collected at 2 dpf (E) and 3 dpf (F), processed for IF, digested, and prepared for FACS. Two-way ANOVA multiple comparison test adjusted *p*-values per each condition at 2 dpf as follow: DMSO:*wild-type versus* DMSO:*kmt2d*^*zy59*^: *p =* 0.7762, DMSO:*wild type versus* DAPT:*wild type*: *p =* 0.9179, DMSO:*wild type versus* DAPT:*kmt2d*^*zy59*^: *p =* 0.9849, DMSO:*kmt2d*^*zy59*^ *versus* DAPT:*wild type*: *p =* 0.9823, DMSO:*kmt2d*^*zy59*^ *versus* DAPT:*kmt2d*^*zy59*^: *p =* 0.9186, DAPT:*wild type versus* DAPT:*kmt2d*^*zy59*^: *p =* 0.9910. Two-way ANOVA multiple comparison test adjusted *p*-values per each condition at 3 dpf as follow: DMSO:*wild type versus* DMSO:*kmt2d*^*zy59*^: *p =* 0.0221 (significant *), DMSO:*wild type versus* DAPT:*wild type*: *p =* 0.5394, DMSO:*wild type versus* DAPT:*kmt2d*^*zy59*^: *p =* 0.9263 (no significant difference, DAPT rescue was to similar control *wild type* values), DMSO:*kmt2d*^*zy59*^ *versus* DAPT:*wild type*: *p =* 0.0012, DMSO:*kmt2d*^*zy59*^ *versus* DAPT:*kmt2d*^*zy59*^: *p =* 0.0039 (significant *, DAPT rescues *kmt2d* mutant Rbpj levels), DAPT:*wild type versus* DAPT:*kmt2d*^*zy59*^: *p =* 0.9839. ANOVA summary results: Interaction F (1, 12) = 4.173, *p* = 0.0637; Treatment F (1, 12) = 19.37, *p* = 0.0009, Genotype F (1, 12) = 9.202, *p* = 0.0104. Values for each data point can be found in S1 Data. dpf, days post fertilization; FACS, fluorescent activated cell sorting; GFP, green fluorescent protein; kdrl, kinase insert domain receptor like.

In agreement with our previous confocal imaging analysis in 5 dpf embryos (Fig 6E and 6F), Rbpj protein levels were increased at 3 dpf in *kmt2d*^*zy59*^ mutants when compared with their *wild-type* siblings (Fig 8A, 8Aʹ, 8C and 8Cʹ). Remarkably, Rbpj protein expression was locally enhanced in the area corresponding to endocardial cells (Fig 8Cʹ, arrow). DAPT-treated *kmt2d*^*zy59*^ embryos showed a substantial general reduction in Rbpj signal (Fig 8D and 8Dʹ) that is particularly distinctive in endocardial cells (Fig 8D and 8Dʹ). In order to quantify this, we proceeded with FACS analysis to measure Rbpj MFI exclusively in endothelial and endocardial cells (GFP positive cells) in individual embryos as described above. Also, to assess the temporal resolution of Rbpj protein levels rescue, we performed this FACS experiment with 2 dpf and 3 dpf embryos.

Our results showed that at 2 dpf, Notch pathway inhibition with DAPT does not affect Rbpj protein levels in any of the analyzed groups, as reflected in similar MFI values (Fig 8E). In contrast, at 3 dpf, Rbpj MFI values were significantly increased in DMSO (control-treated) *kmt2d*^*zy59*^ compared with DMSO-treated wild-type siblings. DAPT treatment resulted in reducing these values to basal Rbpj MFI levels, similar to those observed in *wild-type* control group (Fig 8F, two-way ANOVA multiple comparison test, *n =* 4 per condition, adjusted *p*-values per group; see figure legend for *p-*value details). These results substantiate our findings that Notch signaling is hyperactivated in *kmt2d* mutants, resulting in increased Rbpj expression in endocardial/endothelial cells and further support the hypothesis of a regulatory link between the Notch pathway and Kmt2d.

### Pharmacological inhibition of Notch signaling rescues endothelial patterning and ectopic blood vessel formation in zebrafish *kmt2d* mutants

Our endothelial cell analysis in *kmt2d* mutants revealed an aberrant endothelial cell behavior as early as 2 dpf that precedes disruption of normal AA development (Fig 5A–5C). Moreover, we showed that some endothelial cells located in the region of AA development display tip cell-like phenotypes and generate ectopic blood vessel initiation (Fig 5G–5I). Considering that DAPT treatment was able to partially rescue AA development (Fig 7) and dampen down Notch signaling levels in *kmt2d*^*zy59*^ mutants to wild-type levels (Fig 8), we decided to test whether DAPT would be able to rescue the early endothelial phenotype and reduce ectopic blood vessel sprouting in the AA development area.

We treated *kmt2d*^*zy59*^;*tg(kdrl*:*GFP)* embryos and *wild-type tg(kdrl*:*GFP)* siblings with DAPT or DMSO from 1 dpf to 2 dpf. At 2 dpf, embryos were washed and immediately processed for in vivo time-lapse confocal microscopy. A total number of 24 embryos (12 DMSO controls and 12 DAPT treated) were assigned unique identifiers and imaged laterally, focusing on the AA development region. Images were taken every 20 minutes for a period of 10 hours. Cohorts were imaged from 2 dpf to 2.5 dpf and from 3 dpf to 3.5 dpf. After imaging period, embryos were recovered and individually genotyped.

**Fig 9:**
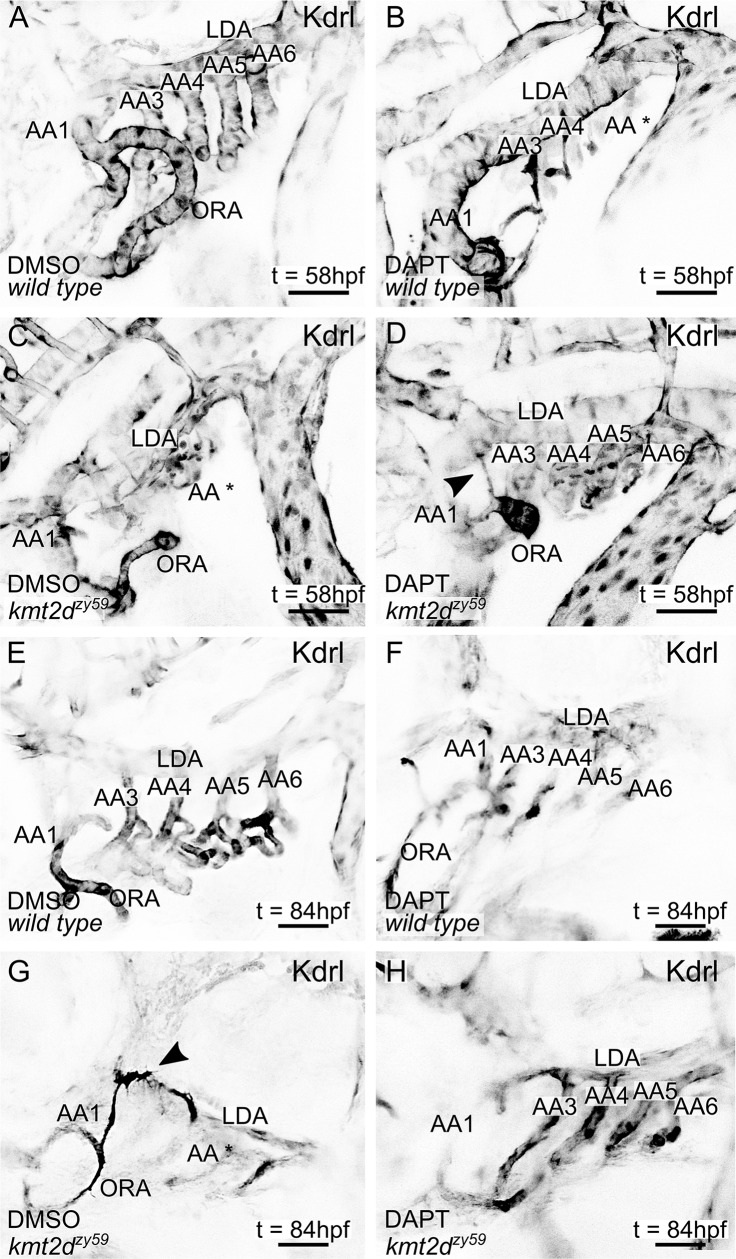
Pharmacological inhibition of Notch signaling rescues early endothelial phenotypes and suppresses ectopic blood vessel formation in zebrafish *kmt2d* mutants. (A–H) Still images (MIP) from time-lapse live imaging performed from 2 dpf to 2.5 dpf (A–D) and 3 dpf to 3.5 dpf (E–H). *wild-type tg(Kdrl*:*GFP)* and *kmt2d*^*zy59*^*;tg(Kdrl*:*GFP)* samples were treated with DMSO (*n* = 12; A, C, E, and G) or DAPT (*n* = 12; B, D, F, and H) from 1 dpf to 2 dpf. After treatment, samples were washed and prepared for in vivo time-lapse imaging. Cranial-lateral view at the level of AA development corresponding to video as follow: A, S7 Video; B, S9 Video; C, S8 Video; D, S10 Video; E, S11 Video; F, S13 Video; G, S12 Video; H, S14 Video. Images were selected at last time points recorded. Videos and images were converted to grayscale and inverted for better visualization. Asterisk (B, C, and G = AA*) denotes abnormal or missing vascular development of AA sprouts at the level of the ventral border of LDA. (scale bars = 50 μm). AA, aortic arch; AA1, mandibular arch; AA2, hyoid arch; AA3, first branchial arch; AA4, second branchial arch; AA5 third branchial arch; AA6, fourth branchial arch; dpf, days post fertilization; hpf, hours post fertilization; kdrl, kinase insert domain receptor like; LDA, lateral ventral aorta; MIP, maximum intensity projection; ORA, opercular artery.

Our results indicated that Notch pathway inhibition with DAPT from 1 dpf to 2 dpf was capable of substantially rescuing AA formation in *kmt2d*^*zy59*^ mutants, including early endothelial cell behavior in the ventral border of the lateral dorsal aorta (LDA) where AA structures emerge (S5–S8 Videos, Fig 9A–9D), followed by substantially rescued formation of AA3 through AA6 by 3.5 dpf (S9–S12 Videos, Fig 9H). Interestingly, observed occasional ectopic blood vessel formation in *kmt2d*^*zy59*^ DAPT-treated mutants at 2 dpf but at lower frequencies than untreated mutants. This indicated that Notch signaling inhibition was not able to completely rescue this phenotype at early stages (Fig 9D, arrowhead). However, this lower frequency of ectopic sprouts did not result in ectopic vessels, because 3 dpf *kmt2d*^*zy59*^ DAPT-treated mutants did not show ectopic blood vessels (S9–S12 Videos, Fig 9H). Moreover, the hyperactive endothelial cell behavior that is observable in the junction between LDA and AA1 in *kmt2d*^*zy59*^ mutant from the DMSO control group was not observed in DAPT-treated *kmt2d*^*zy59*^ mutants (S14 Video, Fig 9G, arrowhead and Fig 9H). These results emphasize the utility of this Kabuki Syndrome model in discovering fine details of cardiovascular development and strengthen the hypothesis of a regulatory link between Notch signaling and Kmt2d in vasculogenesis.

## Discussion

Kabuki Syndrome is a rare multisystemic developmental disorder mainly characterized by postnatal growth deficit, distinct facial features, hearing defects, abnormal neurologic development, immune dysfunction, and CHD, predominantly left-sided defects and coarctation of the aorta [1,3,62]. These cardinal features contribute to KS phenotypes with variable expressivity and different severity degrees. However, patient prognosis and morbidity mainly depends on early diagnosis and treatment of CHD and immune dysfunction [23]. The mechanisms through which KMT2D mutations affects cardiovascular development remain unclear. Development and validation of a genetic KS animal model that provides a strong platform for high-resolution analysis of cardiovascular phenotypes will allow a better understanding of the molecular mechanisms underlying the evolution of KMT2D-related CHD in a KS context.

**Fig 10:**
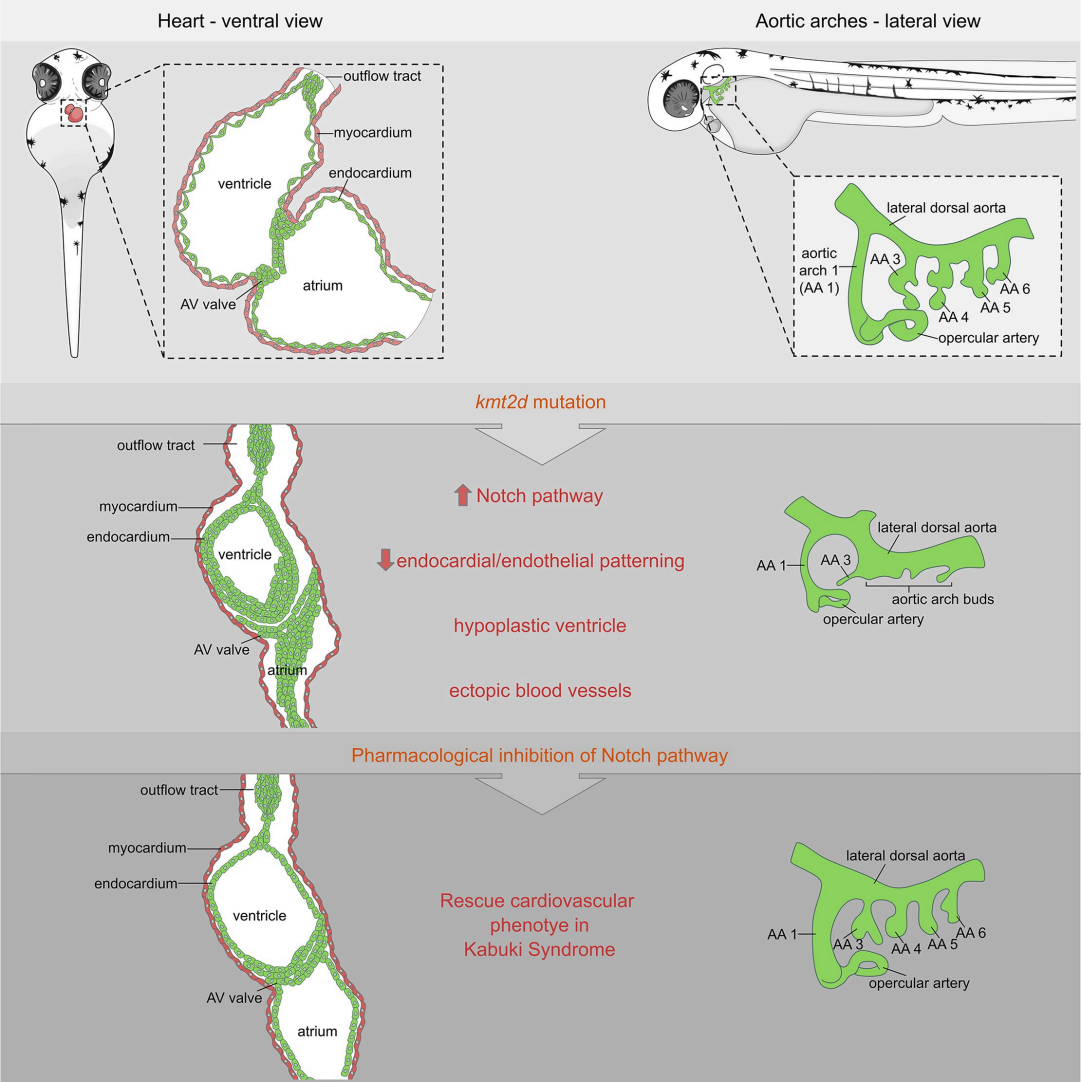
Model summary schematic. Schematic cartoon highlighting cardiovascular defects in our genetic zebrafish model for Kabuki Syndrome. Notch signaling is identified as primary candidate pathway underlying the endothelial/endocardial phenotype. Pharmacological inhibition of Notch signaling was able to rescue the cardiovascular phenotype in KS mutants. AA, aortic arch; AV, atrioventricular.

In this study, we developed a genetic zebrafish model for Kabuki Syndrome that not only recapitulated cardinal phenotypic traits of the human pathology but, most importantly, allowed us to uncover previously unknown cardiovascular defects precipitated by abnormal endothelial/endocardial cell patterning. Through a combination of transcriptome analysis, F0 *kmt2d* mosaic mutants screening, and FACS, we identified Notch signaling in endocardial/endothelial cells as a candidate pathway underlying cardiovascular phenotypes. We also demonstrated that altered Notch pathway signaling was driven at the level of its nuclear transcription factor Rbpj. Importantly, drug inhibition of Notch signaling was able to restore Rbpj levels in endocardial/endothelial cells to normal levels and rescue multiple cardiovascular phenotypes in KS mutants (Fig 10).

Notch pathway is a known regulator of endocardium and vascular patterning during vertebrates’ development [54,63]. Rbpj is the major transcriptional effector of Notch signaling [64]. Recent studies in *Drosophila* demonstrated that KMT2D binds nuclear Notch co-activator complex [65], establishing a strong precedent of a regulatory link between Notch pathway and KMT2D in invertebrates [66]. This report is consistent with our findings that loss of *kmt2d* produces a misbalance of Notch signaling at the level of transcription factor regulation. Our results demonstrate that Rbpj is up-regulated at the transcript and protein level in zebrafish *kmt2d* mutants.

During canonical Notch signaling, ligand-receptor interactions of Notch components drives sequential proteolytic cleavage of the Notch receptor and nuclear translocation of the intracellular domain of Notch (NICD). Once there, NICD binds to the effector protein RBP-J and enables the induction of target genes by recruitment of co-activators in a cell-context dependent manner [67]. However, NICD cleavage also occurs independently of Notch ligand-receptor interaction, thereby providing basal levels of NICD that will bind Rbpj and regulate Notch signaling in a noncanonical fashion [68,69]. In line with this paradigm, our results show that the canonical ligands and receptors of Notch pathway upstream of Rbpj are not affected in *kmt2d* mutants, suggesting that at least part of the response in *kmt2d* mutants is due to a misbalance of noncanonical Notch activity. Additionally, manipulating availability of NICD (canonical and noncanonical) by inhibiting its proteolytic cleavage with DAPT allowed us to rescue the *kmt2d* mutant endocardial phenotype, vascular phenotypes, Rbpj protein levels, and aberrant endothelial cell behavior. Our interpretation is that Kmt2d and Notch signaling operate in cardiovascular development under a stringent Goldilocks principle: hyperactivated Notch signaling (*kmt2d* mutants) or too little Notch signaling (DAPT treatment in wild-type) is deleterious to normal endocardial and endothelial cell development, and that DAPT treatment of *kmt2d* mutants resolves a sufficient “just right” balance of Notch signaling to allow partial rescue of mutant phenotypes. Altogether, these data indicate that the regulatory link between Kmt2d and Rbpj during cardiovascular development occurs in part via noncanonical Notch pathway in a ligand-independent manner. However, under the current paradigm, this hypothesis cannot entirely explain the full extent of our results. Even though the observed vascular mispatterning, ectopic angiogenesis and disorganized endocardium in our *kmt2d* mutants fits with a Notch pathway dysregulation, we do not assume that the underlying molecular mechanisms are the same in each scenario. Further studies are needed in order to fully understand the link of Kmt2d and noncanonical Notch pathway in the context of angiogenesis and vasculogenesis.

Pioneering studies demonstrated the importance of Kmt2d during myocardium development in mammals but did not test a possible role in early endocardium morphogenesis [22]. In this sense, our results in zebrafish *kmt2d* mutants demonstrate for the first time a strong contribution of the endocardium to the hypoplastic ventricle phenotype described here. In contrast to our finding of a fundamental role of Kmt2d during endocardium patterning during zebrafish heart development, our myocardium analysis does not support a myocardial kmt2d mutant phenotype. Thus, we propose that the hypoplastic ventricle and overall cardiac phenotype in Kabuki Syndrome is mainly driven by endocardium mispatterning.

Current knowledge in Kmt2d function is focused on its roles as a H3K4 methyltrasferase [70]. However, not much is known about Kmt2d gene regulatory networks, and it is not known whether Kmt2d has nonhistone substrates. Our transcriptome data provides evidence for a broader functional spectrum of Kmt2d and offers strong gene candidates that we are pursuing in order to comprehend the multisystemic phenotype in zebrafish KS phenotype. Furthermore, our gene set enrichment analyses suggest that genes coding for core ECM proteins have a strong early contribution in *kmt2d*^*zy59*^ mutant’s phenotype. This result suggests that the observed angiogenesis defect in *kmt2d*^*zy59*^ might be a consequence of an abnormal ECM, as previously demonstrated in other disease scenarios such as cancer development [71]. Moreover, our results demonstrate that the observed endothelial/endocardial phenotypes are not not due to cell identity/specification failure nor hypoxia stress but due to defects in the maintenance of cell behaviors.

Overall, these results indicate a novel regulatory link between Kmt2d and Notch pathway in cardiovascular patterning and suggest a possible therapeutic approach for ameliorating KS phenotypic traits of diseases caused by Kmt2d mutations.

## Materials and methods

### Ethics statement

Zebrafish embryos, larvae, and adults were produced, grown, and maintained according to standard protocols approved by the Institutional Animal Care and Use Committee of University of Utah, protocol number 18–05006. For experiments, zebrafish embryos ranging from 1 dpf to 7 dpf were used. Adults were maintained at approximately 5 fish per liter for all experiments in aquarium with controlled light cycle and water temperature at 28°C. Animals were fed 3 times a day. Published strains used in this study include: wild-type AB, *tg(kdrl*:*EGFP)*^*la116*^ [72] and *Tg(EPV*.*Tp1-Mmu*.*Hbb*:*EGFP)*^*um14*^ referred in this manuscript as *tg(tp1*:*EGFP)*^*um14*^ [73].

### Method details

#### Mutants generation and genotyping

Mutants were generated in AB wild-type background. Mutagenesis was induced with CRISPR/Cas9 genome editing tools. Guide RNA was designed and synthesized at the University of Utah Mutation Generation and Detection Core Facility (University of Utah, Salt Lake City, Uath). Guide RNA was designed using as template *kmt2d* sequence available in Genome build Z version 9, Ensembl annotation released version 79. Guide RNA target sequence corresponding to exon 8 is as following: 5ʹ-GTATTGACTGTGGCATGCGA-3ʹ. Genotyping was performed through HRMA [74] using CFX96 Touch Real-Time PCR Detection System. HRMA primer sequences are: forward, 5ʹ-AGT TTT AGC GGT GCC GTG TG-3ʹ; reverse, 5ʹ-CCA CTG TTC AGA GCC AGG AAG-3ʹ. Mutations were confirmed by DNA Sanger sequencing at the DNA Sequencing Core Facility (University of Utah. Salt Lake City, Utah) using the primers: forward, 5ʹ-AGC TTG TAC AGA AGT TTG GCA A-3ʹ; reverse, 5ʹ-GGA AAA GTA CAC TTT AGA AAA CAG C-3ʹ. Multiple alleles were isolated with approximately 5 to 8 independent founders per allele. Mutation was confirmed and phenotypes analyzed in all of them. Lines are maintained as heterozygous in a *kdrl*:*EGFP trans*genic background.

#### RNA extraction for RNA sequencing experiment

RNA was extracted from 50 individual embryos at 1 dpf obtained from a heterozygous by heterozygous(*kmt2d*^*+/zy59*^) cross from 2 parental crosses. We recorded parent ID and order of processing for each embryo to be able to adjust for technical and biological variability. Genomic DNA and total RNA was extracted using Quick-DNA/RNA extraction kit from Zymo (Zymo Research. Irvine, California). After genotyping by HRMA, 6 embryos of each genotype (*wild type*, *heterozygous*, and *mutant*) were selected, with 3 embryos from each of 2 parental crosses. A total of 18 samples were selected, considering processing order and submitted to the High-Throughput Genomics and Bioinformatic Analysis Shared Resource at Huntsman Cancer Institute at the University of Utah (University of Utah. Salt Lake City, Utah). RNA concentrations per sample were on average 8 ng-μL and the RNA integrity Numbers (RINs) were 9 or higher. Libraries were built using Illumina TruSeq Stranded mRNA Library Preparation Kit with polyA selection (Illumina, San Diego, California) and sequencing protocol was HiSeq 125 Cycle Paired-End Sequencing version 4. The 18 samples were multiplexed in 3 sequencing lanes.

#### Alcian Blue/Red alizarin and O-Dianisidine staining

Staining was performed at 4 dpf as previously described by Walker and colleagues [75]. For O-Dianisidine experiment (Millipore Sigma, St. Louis, Missouri. Catalog D9143-5G), 6 dpf embryos were fixed with 4% paraformaldehyde overnight. Fixed embryos were washed in PBS for 3 times and then incubated in the staining buffer (0.6 mg/mL o-dianisidine, 10 mM sodium acetate [pH 5.2], 0.65% hydrogen peroxide, and 40% ethanol) for 15 minutes in the dark.

#### Immunofluorescence

Embryos were fixed over night in 4% paraformaldehyde in PBS with triton 0.4%. Fixed embryos were washed with PBS 1× + triton 0.2% (PBST) 3 times for 15 minutes each in a rotator at room temperature. Pigment was removed, incubating embryos for 15 minutes in a solution containing KOH 0.8%, Tween20 0.1%, H_2_O_2_ 0.9%, and H_2_O to final volume, washed 3 times for 15 minutes each with PBST, and incubated for 1 hour at room temperature with blocking solution (PBST + 5% goat serum). Primary antibody incubation was performed over night at 4°C. Primary antibody was removed and samples were washed with PBST 3 times for 30 minutes each. Samples were incubated with secondary antibody over night at 4°C.

Antibody dilutions were performed in PBST with 1% goat serum.

After secondary antibody incubation, samples were washed with PBST 3 times for 30 minutes each and rinsed for 15 minutes with increasing concentrations of glycerol (30%, 50%, and 80%) in PBS 1×. Stained samples were mounted in low melt agarose at 1% for confocal imaging.

Primary antibody concentration was established following manufacturer guidelines; secondary antibody concentration was 1:500 in all cases. Antibodies are detailed in the Key Resource Table. Anti-GFP antibody was used to enhance endogenous *trans*genic fluorescence detection in *tg(kdrl*:*GFP)* line. Nuclear staining is not shown in processed images, but it was performed for cell quantification experiments. DAPI was used in a concentration of 1 mg.ml^−1^ and applied during secondary antibody incubation step.

#### Single-embryo FACS

Embryos were fixed and processed for immunofluorescence as described in the previous section. After secondary antibody incubation and washes, samples were distributed placing 1 single embryo per 1.5 mL tube. PBST was completely removed and 1 mL of prewarmed TrypLE (1×) solution (ThermoFisher Scientific. Waltham, Massachusetts) was added in each tube. Samples were incubated for 1.5 hours at 37°C. Pipetting every 10 minutes was performed to aid in dissociation. At the end of 1.5 hours, solution was checked for cell aggregates. TrypLE reaction was stopped by adding CaCl_2_ to a final concentration of 1 mM and fetal bovine serum (ThermoFisher Scientific. Waltham, Massachusetts) to 10%. Each sample was filtered through a 40 μm nylon mesh filter (Fisher 22363547; ThermoFisher Scientific. Waltham, Massachusetts) into a 50 mL tube. Filtered samples were spun down at 2,500 RPM for 10 minutes at 4°C. Supernatant was discarded and cells of each individual embryo were resuspended in 300 μL of PBS 1×.

For cell cycle profiling, cells were resuspended in DAPI staining solution (Triton 0.1%, PBS 1×, DAPI 1 mg.ml^−1^). For accurate sample gating, controls in each experiment included: samples without fluorescence (for excluding yolk autofluorescence), samples without fluorescence with secondary antibody incubation only (for excluding secondary antibody unspecificity), samples with DAPI, samples with fluorescence of interest, and samples with DAPI and fluorescence of interest. Samples were processed for flow cytometry analysis on a BD FACS Canto and Propel Labs Avalon Cell Sorter at the University of Utah Flow Cytometry Facility. FACS data was analyzed with FlowJo (version 9) software (https://www.flowjo.com/).

#### Confocal images acquisition, processing, and analysis

Images were acquired in Zeiss LSM880 with Airyscan fast module following optimal parameters for superresolution (SR) mode acquisition. Image acquisition was performed keeping the same laser parameters for the channel of interests (channel to be measured/quantified). Acquisition parameters corresponding to context markers were enhanced as required for image clarity. Output files were processed for Airyscan processing with Zen Black software keeping default parameters. Output files (.czi) were processed with Fiji (ImageJ) to build maximum intensity projections for slides of interests or the complete data set.

Imaris software (version 9.2; Bitplane. https://imaris.oxinst.com/) was used to reconstruct 3D microscopy images, measure heart ventricle volume, cell count, and cell morphology numbers. The Imaris *surfaces* feature was used to analyze the volume of the ventricle by generating a surface in the lumen of each ventricle. Endocardial cells were used as a limit so the space delimited by them was considered ventricle lumen and measured. Cell numbers were gathered by creating a surface containing the channel corresponding to myocardium, endocardium, or Notch positive cells, and the channel corresponding to DAPI was masked in each tissue/cell specific surface. The *measurement points* tool was used to count number of nuclei in each surface.

Myocardial cells area and circularity were measured using the *surfaces* tool in manual mode. Alcama antibody was used a reference marker for myocardial cell limits. The *clipping plane* feature of Imaris was used to visualize each cell and validate the surface created. Details of this method can be found in S3 Fig legend.

#### Time-lapse experiments

Time-lapse experiments were performed in embryos from a *kmt2d*^*zy59*^;*tg(kdrl*:*GFP)* het by het cross at 2 dpf, 3 dpf, and 4 dpf. In each case, genotype was confirmed at the end of the experiment by phenotype screening and HRMA. Samples were placed in embryo water with N-Phenylthiourea 1× (PTU) for avoiding pigment formation and anaesthetized with MS-222 (150 mg/L^−1^). Samples were embedded in 0.6% low melt agarose with MS-222 in embryo water.

Acquisition was performed in Zeiss LSM880 with Airyscan fast module using 10× dry objective and default *optimum* parameters. Parameters of acquired data as follow: 16 bit depth image, 1.8 zoom, 1176 × 1176 pixels image size and minimum z-stack interval for capturing development of AA (approximately 467.56 × 467.56 × 2.88 μm). Images were acquired every 20 minutes for a length of 12 hours. After acquisition period, samples were recovered for genotyping and/or immunofluorescence and SR confocal imaging.

#### F0 experiment—kmt2d mosaic analysis

For analyzing the effects of *kmt2d* mutation in a Notch reporter background, the *kmt2* guide RNA/Cas9 (described in the section Mutant generation) was injected in *tg(tp1*:*EGFP)*^*um14*^ embryos. Approximately 100 embryos per clutch were injected, and 40 of them were processed for *kmt2d* mutation validation though HRMA. Injected embryos showed a range of phenotype from mild to strong effects. A total of 7 embryos per condition (injected–not injected) were selected based on their gross morphology similarity with class II *kmt2d*^*zy59*^ mutants (Fig 1B).

#### qPCR experiment

A total of 3 dpf wild-type/heterozygous and *kmt2*^*zy59*^ mutant embryos were lysed in TRI Reagent (Zymo) and processed for RNA using a Zymo Quick-RNA kit. The total RNA was used for cDNA synthesis with BioRad iScript 5× master mix (BioRad Laboratories. Hercules, California. Catalog 4106228). Subsequent cDNA was used for qPCR reactions (CFX96 Touch Real-Time PCR Detection System) with the following gene primers: *notch1b*, *rbpja*, *hes1*, *and elfα*. Primer and probe sequences are listed in the Key Resources table (S3 Table). ΔCt calculations were normalized to *elfα* expression levels and control sibling expression levels. The number of samples used was *N* = 4 per genotype with 2 technical replicates per gene and per genotype assessed.

#### Pharmacological treatment

For Notch pathway inhibition, embryos from a *kmt2d*^*zy59*^;*tg(kdrl*:*GFP)* het by het cross were treated with 50 μM DAPT (Selleckchem. Houston, Texas. Catalog S2215) from 1 dpf to 2 dpf. After treatment, embryos were washed with fresh embryo water and immediately processed for time-lapse experiment or maintained until 3 dpf or 5 dpf for immunofluorescence sample processing depending on the experiment. After fixation, DAPT-treated embryos were tail-clipped for DNA extraction and genotypification by HRMA. All treatments, including controls, were as follows: *wild-type* DMSO, *wild-type* DAPT, *kmt2d*^*zy59*^ DMSO, and *kmt2d*^*zy59*^ DAPT.

For hypoxia induction, *tg(kdrl*:*GFP)* and *kmt2d*^*zy59*^*;tg(kdrl*:*GFP)* embryos were treated from 3 dpf to 4 dpf with 100 μM DMOG (HIF-Hydroxylase Inhibitor). After treatment, embryos were washed and processed for IF and confocal imaging. DMSO was used as control for drug solvent in mutant and wild-type genotypes.

### Quantification and statistical analysis

#### General statistical analysis

Sample sizes were chosen based on previous publications and are defined in each figure legend. No sample was excluded from the analysis. Unless explicitly expressed in the text or figure legend (Fig 7F and 7H, S3C Fig), the experiments were not randomized, and the investigators were not blinded to allocation during experiments and outcome assessment. All statistical values are displayed as mean ± standard deviation with exception of S3A and S3C Fig were 25th percentile, 50th percentile, and 75th percentile are shown. Sample sizes, statistical test, and *p*-values are indicated in the figures or figure legends. Statistical significance was assigned at *p* < 0.05. Statistical tests were performed using Prism 7 software (GraphPad, https://www.graphpad.com/scientific-software/prism/) and R package.

#### RNA differential expression analysis

Transcriptome was aligned with STAR [76] to Genome build Z version 9, Ensembl annotation released version 79. In this study, differential expression analysis was performed between 6 wild-type versus 6 mutant samples with DESeq2 [77] using the negative binomial likelihood ratio test. Heterozygous samples were excluded from this analysis. An initial cutoff of 5% false discovery rate was set.

#### GSEA

To perform GSEA analysis, we used the differential expression analysis gene list ranked by *p*-values. We then filtered the list to obtain genes that have one-to-one orthology to human genes as specified in the Ensembl Compara database that were retrieved with BiomaRt package [78]. Gene sets obtained from the Molecular Signatures Database (MSigDB). The resulting list was additionally filtered using a mean normalized count cutoff of 5. The number of resulting genes identifiers analyzed was 9,128 out of 33,737.

GSEA preranked analysis (10,000,000 permutations, minimum term size of 15, maximum term size of 500) was then performed using fgsea package (https://github.com/ctlab/fgsea/)[79]

The analysis was performed independently with 8 selected gene sets and subsets from MSigDB: H: hallmark gene sets, CP: Canonical pathways, CP: KEGG: KEGG gene sets, CP:REACTOME: Reactome gene sets, BP: GO biological process, CC: GO cellular component, MF: GO molecular function, C7: immunologic signatures. Results cutoff was set at 5% FDR and plotted using R programming interface.

## Supporting information

S1 Fig Kmt2d protein expression. (A-B) Kmt2d antibody technical control. Confocal images of 5 dpf zebrafish embryo in a lateral view. IF was performed against Kmt2d (red and black) and α-ac-tub (yellow) as context marker. Kmt2d antibody specificity for zebrafish was assessed through IF negative control. (A) Positive control of Kmt2d antibody. (B) Kmt2d negative control. Samples were processed in parallel to the positive controls. Primary antibody incubation was omitted, and samples were incubated with secondary antibodies. (Aʹ–Bʹ) Kmt2d channel was selected, set as grayscale, and the look-up table was inverted in order to enhance contrast. (C–E) Kmt2d protein expression time course. Confocal images of ventral views of zebrafish *tg(kdrl*:*GFP)* embryos at 17 hpf (C–C"), 2 dpf (D–D"), and 3 dpf (E–E"). Immunofluorescence was performed against Kmt2d (red) and GFP (Kdrl, green) as context marker. (C, D, and E) Merge for Kmt2d and Kdrl. (Cʹ, Dʹ, and Eʹ) Channel for Kmt2d (red). White dashed line delineates the heart (Dʹ and Eʹ). Images were processed as MIP. (F–H) Kmt2d null mutant validation. Confocal images of 5 dpf zebrafish embryos in a ventral view. Images were processed as MIPs. IF was performed against Kmt2d (red and black) and myosin heavy chain (MF20, green) as context marker. Samples were genotyped by HRMA after image acquisition. (F) Homozygous *wild type*, (G) heterozygous, and (H) homozygous mutant. Note the lack of Kmt2 protein expression confirming *kmt2d*^*zy59*^ as null mutant. (Fʹ–Hʹ) Kmt2d channel was selected, set as grayscale, and the look-up table was inverted in order to enhance contrast. dpf, days post fertilization; hpf, hours post fertilization; IF, immunofluorescence; kmt2d, Histone-lysine N-methyltransderase 2D; MF20, Myosin Heavy Chain Antibody; MIP, maximum intensity projection; α-ac-tub, alpha acetylated tubulin.
(TIFF)

S2 Fig *kmt2d* mutant phenotype at 4dpf. (A–C) Lateral view of zebrafish *wild-type* sibling embryo (A) and *kmt2d*^*zy59*^ mutants (B, C) at 4 dpf. At 4 dpf *kmt2d*^*zy59*^ embryos develop general body edema that increases gradually at later stages. (D–F) Alcian blue/ Alizarin red staining in 2 additional mutant alleles. dpf, days post fertilization.
(TIFF)

S3 Fig Analysis of myocardial cell morphology, apoptosis, and heart rate in *wild-type* siblings and *kmt2d^zy59^* mutants. (A) Myocardial cell shape analysis in *kmt2d*^*zy59*^ mutants at 3 dpf. *Wild-type* sibling and *kmt2d*^*zy59*^ mutant embryos were processed for IF against Alcama for cell-cell boundaries and myosine heavy chain (MF20) for myocardium context. Z-stacks were analyzed with Imaris software. Area and circularity were measured in 5 different cells from the outer curvature of the ventricle. Averaged values are plotted. There is no significant difference in cardiomyocytes shape in wild-type samples versus mutants. *t* Test, *p* < 0.583 n.s., t = 0.59, dF = 5 for area and *p* < 0.946 n.s., t = 0.71, dF = 5 for circularity. (B) Apoptosis analysis in *wild-type* versus *kmt2d*^*zy59*^ mutant heart. Confocal images of *wild-type* sibling and *kmt2d*^*zy59*^ at 5 dpf. The heart was acquired from a ventral view. IF was performed against active-caspase3 for apoptosis evaluation and Alcama and MF20 as context markers. Arrows and arrowheads point to apoptotic cells. (C) Heart rate comparison in *wild-type* siblings versus *kmt2d*^*zy59*^ mutants at 1, 2, 3, and 4 dpf. Embryos were placed individually in a 96-well plate. Measurements were performed at each time point to the same animal subject every time in a blind fashion until day 3 through 4, when the phenotype was apparent. Heart beat count was performed for 15 seconds without anesthetic to avoid any secondary effects that could impact heart rate. Heart rate values were adjusted according to the ANOVA model, for both experiment and time points variability *p* = 0.000264, F (1,76) = 14.647. dpf, days post fertilization; IF, immunofluorescence; MF20, Myosin Heavy Chain Antibody.
(TIFF)

S4 Fig Vascular network analysis in *wild-type* siblings and *kmt2d^zy59^* mutants. (A–D) *o-dianisidine* staining for assessing vasculature integrity in *kmt2d*^*zy59*^ and *wild-type* siblings at 6 dpf. Lateral views (A, B) and cranial-ventral views (C, D) of *wild-type* sibling (A, C) and *kmt2d*^*zy59*^ mutant (B, D) at 6 dpf. White arrowheads indicate blood aggregates in the region of AA and head. Scale bar = 100 μm. (E–H) Vascular development at 3 dpf and 4 dpf in *wild-type* sibling versus *kmt2d*^*zy59*^ mutant embryos. Confocal images of cranio-lateral views at 3 dpf (E, F) and 4 dpf (G, H) in *wild-type* (E–E", G, G") and mutant (F–F", H, H") embryos. IF was performed against GFP, for enhancing Kdrl:GFP *trans*genic signal, HuC/D and α-acetylated tubulin as context markers. AA, aortic arch; dpf, days post fertilization; GFP, green fluorescent protein; IF, mmunofluorescence; kdrl, kinase insert domain receptor like.
(TIFF)

S5 Fig Vascular development in induced hypoxia conditions in *wild-type* and *kmt2d^zy59^* embryos. Confocal images show cranial-lateral view of vasculature in *wild-type tg(kdrl*:*GFP)* sibling (A) and *kmt2d*^*zy59*^;*tg(kdrl*:*GFP)* mutants at 4 dpf. (A–B) DMSO controls for both wild-type sibling and *kmt2d* mutant. (C–D) DMOG treated embryos. Treatment was performed from 3 to 4 dpf. White arrowheads indicate hypoxia-induced blood vessel sprouting. White arrows (B and D) indicate *kmt2d* mutation-dependent ectopic blood vessel formation in both DMSO control and DMOG treated embryos. dpf, days post fertilization.
(TIFF)

S6 Fig F0 *kmt2d* mosaic mutants phenotype validation. CRISPR/Cas9 injection against kmt2d produces comparable phenotype to the observed in germline mutants (arrows and arrowheads). A, C, E, Noninjected controls. B, D, F, injected embryos. E, F, confocal images of noninjected controls and kmt2d injected embryos. IF was performed for Myosin heavy chain (M20, green), Alcama (zn5, red), and Myosin heavy chain, atrium specific (S46, red) as general myocardium morphology markers. Dashed white line highlights hypoplastic heart as a consequence of mutated *kmt2d* through CRISPR injection. F0, filial 0; IF, immunofluorescence; kmt2d, Histone-lysine N-methyltransderase 2D; M20, Myosin Heavy Chain Antibody.
(TIFF)

S7 Fig Proliferation assay for validating drug rescue phenotype. (A–D) Confocal images of *wild-type* sibling (A, B) and *kmt2d*^*zy59*^ mutant (C, D) embryos at 5 dpf. DMSO as solvent control (A, C) and DAPT for Notch signaling inhibition (B, D) were applied to embryos of indicated genotypes. IF against GFP was performed to enhance Kdrl:GFP trangenic signal (endothelium and endocardium). MF20 (myosin) was use as context marker for muscle. phH3 (cell proliferation) marks mitotic cells. Note the increased phH3 signal in cardiovascular area in *kmt2d* mutants after DAPT treatment (D). dpf, days post fertilization; GFP, green fluorescent protein; IF, immunofluorescence; kdrl, kinase insert domain receptor like; MF20, Myosin Heavy Chain Antibody; phH3, phospho Histone 3
(TIFF)

S8 Fig Proliferation assay in endothelium/endocardium at 3 dpf. (A–B) Confocal images of *wild-type* sibling (A) and *kmt2d*^*zy59*^ mutant (B) embryos at 3 dpf. IF against GFP was perform to enhance Kdrl:GFP trangenic signal (endothelium and endocardium). pH3 (cell proliferation) marks mitotic cells. Quantification of pH3 was performed exclusively in endothelial and endocardial cells using Imaris (version 9.2) software. dpf, days post fertilization; GFP, green fluorescent protein; IF, immunofluorescence; kdrl, kinase insert domain receptor like.
(TIFF)

S1 Data (XLSX)

S1 Table Text-mining of top 50 differentially regulated genes. Top 50 gene candidate at a 5% FDR (base mean, log2 fold change and *p* adjusted values are specified). Manual text-mining was performed for each candidate. Categories were established considering available information for cell compartment, biological function, region of expression and mammal orthologs data. FDR, false discovery rate.
(DOCX)

S2 Table GSEA. Analysis was performed by converting zebrafish gene names to human gene names using exclusively genes with a one-to-one ortholog relationship. The number of resulting genes identifiers analyzed was 9,128 out of 33,737 (Genome build Z version 9, Ensembl annotation released version 79). Adjusted *p*-values were calculated per category. NES of gene sets with a FDR of 5% (blue dots) and 15% (purple dots) were plotted to summarized GSEA results. FDR, false discovery rate; GSEA, gene set enrichment analysis; NES, Normalized Enrichment Score.
(DOCX)

S3 Table Key Resources table. (DOCX)

S1 Video Three-dimensional render of cardiovascular patterning of *wild-type* sibling at 5 dpf. dpf,days post fertilization
(MOV)

S2 Video Three-dimensional render of cardiovascular patterning of *kmt2d^zy59^* mutant at 5 dpf. dpf, days post fertilization
(MOV)

S3 Video Twenty embryos from a *kmt2d^+/−^* by *kmt2d^+/−^* cross were mounted in 0.6% agarose for overnight time-lapse imaging. Lateral view with head toward viewer’s left. Genotypes corresponding to *wild type/heterozygous* or *mutants* were assigned at the end of the experiment at 3 dpf when phenotype was evident. One wild-type embryo (S3 Video) and one *kmt2d*^*zy59*^ mutant embryo (S4 Video) are shown as examples. Note A3 through AA6 develop and extend almost overlapping with ORA in *wild-type* embryo (S3 Video). The AA growth occurs in a cranio-caudal direction with AA3 development before AA6. In contrast, *kmt2d* mutant embryo does not develop normal AA primary sprouting. Endothelial cell sprouting and extension were hyperactive, particularly in the more ventral-caudal region of the LDA, where AA6 normally develops. Moreover, AA6 shows an abnormal primary sprout before the rest of AA (S4 Video). AA, aortic arch; dpf, days post fertilization; LDA, lateral dorsal aorta; ORA, opercular artery.
(MOV)

S4 Video Twenty embryos from a *kmt2d^+/−^* by *kmt2d^+/−^* cross were mounted in 0.6% agarose for overnight time-lapse imaging. Lateral view with head toward viewer’s left. Genotypes corresponding to *wild type/heterozygous* or *mutants* were assigned at the end of the experiment at 3 dpf when phenotype was evident. One wild-type embryo (S3 Video) and one *kmt2d*^*zy59*^ mutant embryo (S4 Video) are shown as examples. Note A3 through AA6 develop and extend almost overlapping with ORA in *wild-type* embryo (S3 Video). The AA growth occurs in a cranio-caudal direction with AA3 development before AA6. In contrast, *kmt2d* mutant embryo does not develop normal AA primary sprouting. Endothelial cell sprouting and extension were hyperactive, particularly in the more ventral-caudal region of the LDA, were AA6 normally develops. Moreover, AA6 shows an abnormal primary sprout before the rest of AA (S4 Video). AA, aortic arch; dpf, days post fertilization; LDA, lateral dorsal aorta; ORA, opercular artery.
(MOV)

S5 Video Embryos from a *kmt2d^+/−^* by *kmt2d^+/−^* cross at 3 dpf (when phenotype of *kmt2d* mutants is evident) were mounted in 0.6% agarose for overnight time-lapse imaging. Lateral view with head toward viewer’s left. One wild-type embryo (S5 Video) and one *kmt2d*^*zy59*^ mutant embryo (S6 Video) are shown as examples. For detailed description please review main text. dpf, days post fertilization.
(MOV)

S6 Video Embryos from a *kmt2d^+/−^* by *kmt2d^+/−^* cross at 3 dpf (when phenotype of *kmt2d* mutants is evident) were mounted in 0.6% agarose for overnight time-lapse imaging. Lateral view with head toward viewer’s left. One wild-type embryo (S5 Video) and one *kmt2d*^*zy59*^ mutant embryo (S6 Video) are shown as examples. For detailed description please review main text. dpf, days post fertilization.
(MOV)

S7 Video Embryos from a *kmt2d^+/−^* by *kmt2d^+/−^* cross were treated with DMSO (control) or DAPT (treatment for Notch inhibition). At 2 dpf, 24 embryos were randomly selected (12 from control and 12 from treatment), assigned unique identifiers, and mounted in 0.6% agarose for overnight time-lapse imaging (10 hours time-lapse). Lateral view with head toward viewer’s left. Genotypes corresponding to *wild type/heterozygous* or *mutants* were assigned at the end of the experiment by DNA extraction and HRMA. One wild-type embryo (DMSO: S7 Video, DAPT: S9 Video) and one *kmt2d*^*zy59*^ mutant embryo (DMSO: S8 Video, DAPT: S10 Video) per treatment are shown as examples. For references, review Fig 9A–9D. dpf, days post fertilization; HRMA, High Resolution Melt Analysis.
(MOV)

S8 Video Embryos from a *kmt2d^+/−^* by *kmt2d^+/−^* cross were treated with DMSO (control) or DAPT (treatment for Notch inhibition). At 2 dpf, 24 embryos were randomly selected (12 from control and 12 from treatment), assigned unique identifiers and mounted in 0.6% agarose for overnight time-lapse imaging (10 hours time-lapse). Lateral view with head toward viewer’s left. Genotypes corresponding to *wild type/heterozygous* or *mutants* were assigned at the end of the experiment by DNA extraction and HRMA. One wild-type embryo (DMSO: S7 Video, DAPT: S9 Video) and one *kmt2d*^*zy59*^ mutant embryo (DMSO: S8 Video, DAPT: S10 Video) per treatment are shown as examples. For references, review Fig 9A–9D. dpf, days post fertilization; HRMA, High Resolution Melt Analysis.
(MOV)

S9 Video Embryos from a *kmt2d^+/−^* by *kmt2d^+/−^* cross were treated with DMSO (control) or DAPT (treatment for Notch inhibition). At 2 dpf, 24 embryos were randomly selected (12 from control and 12 from treatment), assigned unique identifiers and mounted in 0.6% agarose for overnight time-lapse imaging (10 hours time-lapse). Lateral view with head toward viewer’s left. Genotypes corresponding to *wild type/heterozygous* or *mutants* were assigned at the end of the experiment by DNA extraction and HRMA. One wild-type embryo (DMSO: S7 Video, DAPT: S9 Video) and one *kmt2d*^*zy59*^ mutant embryo (DMSO: S8 Video, DAPT: S10 Video) per treatment are shown as examples. For references, review Fig 9A–9D. dpf, days post fertilization; HRMA, High Resolution Melt Analysis.
(MOV)

S10 Video Embryos from a *kmt2d^+/−^* by *kmt2d^+/−^* cross were treated with DMSO (control) or DAPT (treatment for Notch inhibition). At 2 dpf, 24 embryos were randomly selected (12 from control and 12 from treatment), assigned unique identifiers and mounted in 0.6% agarose for overnight time-lapse imaging (10 hours time-lapse). Lateral view with head toward viewer’s left. Genotypes corresponding to *wild type/heterozygous* or *mutants* were assigned at the end of the experiment by DNA extraction and HRMA. One wild-type embryo (DMSO: S7 Video, DAPT: S9 Video) and one *kmt2d*^*zy59*^ mutant embryo (DMSO: S8 Video, DAPT: S10 Video) per treatment are shown as examples. For references, review Fig 9A–9D. dpf, days post fertilization; HRMA, High Resolution Melt Analysis.
(MOV)

S11 Video Embryos from a *kmt2d^+/−^* by *kmt2d^+/−^* cross were treated with DMSO (control) or DAPT (treatment for Notch inhibition). At 3 dpf, 24 embryos were randomly selected (12 from control and 12 from treatment), assigned unique identifiers, and mounted in 0.6% agarose for overnight time-lapse imaging (10 hours time-lapse). Lateral view with head toward viewer’s left. Genotypes corresponding to *wild type/heterozygous* or *mutants* were assigned at the end of the experiment by DNA extraction and HRMA. One wild-type embryo (DMSO: S11 Video, DAPT: S13 Video) and one *kmt2d*^*zy59*^ mutant embryo (DMSO: S12 Video, DAPT: S14 Video) per treatment are shown as examples. For references, review Fig 9E–9H. dpf, days post fertilization; HRMA, High Resolution Melt Analysis.
(MOV)

S12 Video Embryos from a *kmt2d^+/−^* by *kmt2d^+/−^* cross were treated with DMSO (control) or DAPT (treatment for Notch inhibition). At 3 dpf, 24 embryos were randomly selected (12 from control and 12 from treatment), assigned unique identifiers, and mounted in 0.6% agarose for overnight time-lapse imaging (10 hours time-lapse). Lateral view with head toward viewer’s left. Genotypes corresponding to *wild type/heterozygous* or *mutants* were assigned at the end of the experiment by DNA extraction and HRMA. One wild-type embryo (DMSO: S11 Video, DAPT: S13 Video) and one *kmt2d*^*zy59*^ mutant embryo (DMSO: S12 Video, DAPT: S14 Video) per treatment are shown as examples. For references, review Fig 9E–9H. dpf, days post fertilization; HRMA, High Resolution Melt Analysis.
(MOV)

S13 Video Embryos from a *kmt2d^+/−^* by *kmt2d^+/−^* cross were treated with DMSO (control) or DAPT (treatment for Notch inhibition). At 3 dpf, 24 embryos were randomly selected (12 from control and 12 from treatment), assigned unique identifiers and mounted in 0.6% agarose for overnight time-lapse imaging (10 hours time-lapse). Lateral view with head toward viewer’s left. Genotypes corresponding to *wild type/heterozygous* or *mutants* were assigned at the end of the experiment by DNA extraction and HRMA. One wild-type embryo (DMSO: S11 Video, DAPT: S13 Video) and one *kmt2d*^*zy59*^ mutant embryo (DMSO: S12 Video, DAPT: S14 Video) per treatment are shown as examples. For references, review Fig 9E–9H. dpf, days post fertilization; HRMA, High Resolution Melt Analysis.
(MOV)

S14 Video Embryos from a *kmt2d^+/−^* by *kmt2d^+/−^* cross were treated with DMSO (control) or DAPT (treatment for Notch inhibition). At 3 dpf, 24 embryos were randomly selected (12 from control and 12 from treatment), assigned unique identifiers and mounted in 0.6% agarose for overnight time-lapse imaging (10 hours time-lapse). Lateral view with head toward viewer’s left. Genotypes corresponding to *wild type/heterozygous* or *mutants* were assigned at the end of the experiment by DNA extraction and HRMA. One wild-type embryo (DMSO: S11 Video, DAPT: S13 Video) and one *kmt2d*^*zy59*^ mutant embryo (DMSO: S12 Video, DAPT: S14 Video) per treatment are shown as examples. For references, review Fig 9E–9H. dpf, days post fertilization; HRMA, High Resolution Melt Analysis.
(MOV)

## Abbreviations

AA: aortic arch
AA1: mandibular arch
AA2: hyoid arch
AA3: first branchial arch
AA4: second branchial arch
AA5: third branchial arch
AA6: fourth branchial arch
ACeV: anterior cephalic vein
ADAM: containing a disintegrin and metalloprotease
AV: atrioventricular
avc: atrioventricular canal
α-ac-tub: alpha acetylated tubulin
BRAF: v-raf murine sarcoma viral oncogene homolog B1
CHD: congenital heart defect
Ct: cycle threshold
ΔΔCt: delta-delta cycle threshold
CtA: central artery
DCV: dorsal ciliary vein
DLV: dorsal longitudinal vein
DMOG: dimethyloxalyglycineinduced
dpf: days post fertilization
EC: endocardial and/or endothelial cells
ECM: extracellular matrix
ey: eye
ER: endoplasmic reticulum
F0: filial 0
FACS: fluorescent activated cell sorting
FDR: false discovery rate
G0: generation 0
GFP: green fluorescent protein
GO: gene ontology
GSEA: gene set enrichment analysis
HA: hypobranchial artery
het: heterozygous
HRMA: High Resolution Melt Analysis
HMG: High Mobility Group domain
H3K4: lysine 4 of histone 3
ie: inner ear
IF: immunofluorescence
IOC: inner optic circle
kdrl: kinase insert domain receptor like
KMT2D: Histone-Lysine N-Methyltransferase 2D
LDA: lateral dorsal aorta
LRT: Likelihood Ratio Test
MAPK: Mitogen-activated protein kinase
MFI: Median Fluorescence Intensity
MF20: Myosin Heavy Chain Antibody
MIP: maximum intensity projection
mo: mouth
MsigDB: Molecular Signatures Database
MsV: mesencephalic vein
NCA: nasal ciliary artery
NES: Normalized Enrichment Score
NICD: Notch intracellular domain
n.s.: not significant
OMIM: Online Mendelian Inheritance in Man
ORA: opercular artery
OV: optic vein
oft: outflow tract
PHD: Plant Homeo Domain
pH3: phospho Histone 3
RAS: retrovirus-associated DNA sequence
RIN: RNA integrity number
RNA-seq: RNA sequencing
RT-qPCR: reverse transcription-quantitative polymerase chain reaction
SET: Su-Enhancer-of-zeste and Trithorax
sgRNA: single-guide RNA
SR: superresolution
VA: ventral aorta
ventr/ven: ventricle
