# Supplementary figures and images for: Inhibition of Notch signaling rescues cardiovascular development in Kabuki Syndrome
Source: PLoS Biol. 2019 Sep 3;17(9):e3000087. doi: 10.1371/journal.pbio.3000087 (PMC6743796; doi:10.1371/journal.pbio.3000087)

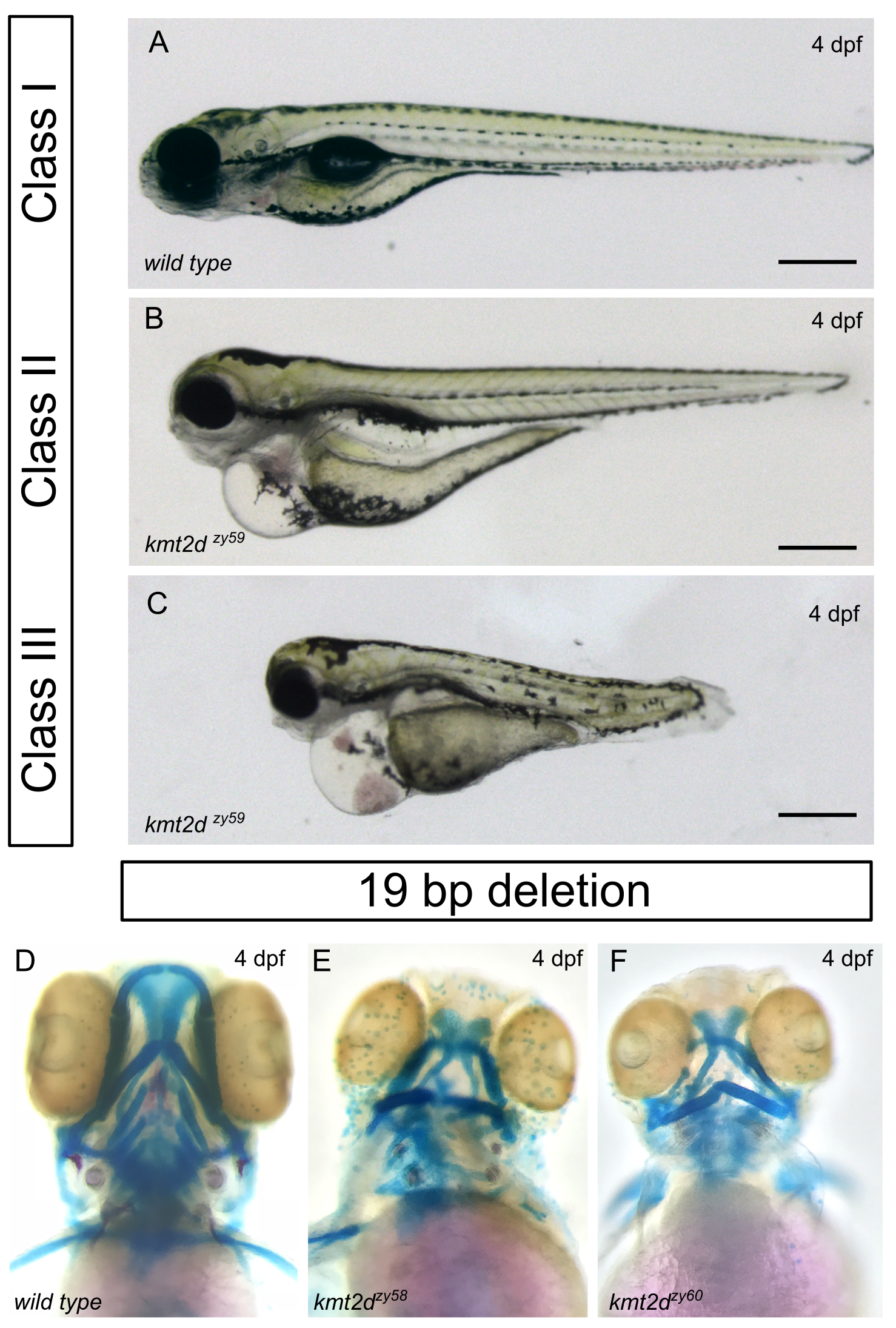

Supplement: S2 Fig — (A–C) Lateral view of zebrafish wild-type sibling embryo (A) and kmt2dzy59 mutants (B, C) at 4 dpf. At 4 dpf kmt2dzy59 embryos develop general body edema that increases gradually at later stages. (D–F) Alcian blue/ Alizarin red staining in 2 additional mutant alleles. dpf, days post fertilization. (TIFF) [file pbio.3000087.s002.tiff]

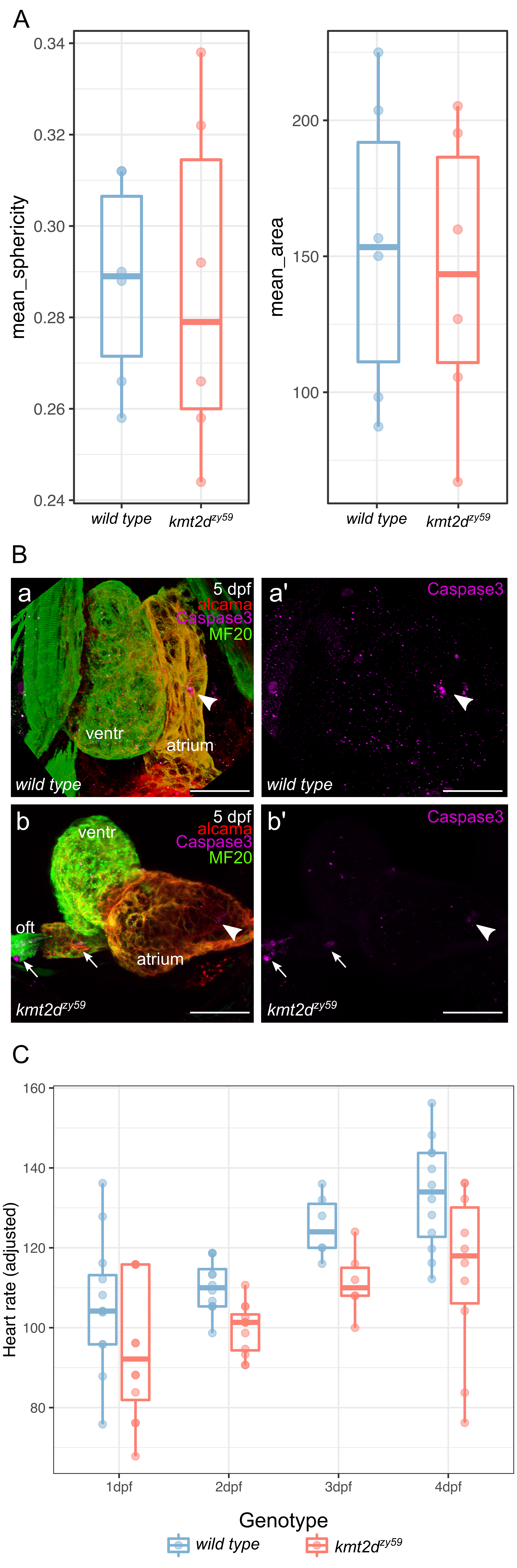

Supplement: S3 Fig — (A) Myocardial cell shape analysis in kmt2dzy59 mutants at 3 dpf. Wild-type sibling and kmt2dzy59 mutant embryos were processed for IF against Alcama for cell-cell boundaries and myosine heavy chain (MF20) for myocardium context. Z-stacks were analyzed with Imaris software. Area and circularity were measured in 5 different cells from the outer curvature of the ventricle. Averaged values are plotted. There is no significant difference in cardiomyocytes shape in wild-type samples versus mutants. t Test, p < 0.583 n.s., t = 0.59, dF = 5 for area and p < 0.946 n.s., t = 0.71, dF = 5 for circularity. (B) Apoptosis analysis in wild-type versus kmt2dzy59 mutant heart. Confocal images of wild-type sibling and kmt2dzy59 at 5 dpf. The heart was acquired from a ventral view. IF was performed against active-caspase3 for apoptosis evaluation and Alcama and MF20 as context markers. Arrows and arrowheads point to apoptotic cells. (C) Heart rate comparison in wild-type siblings versus kmt2dzy59 mutants at 1, 2, 3, and 4 dpf. Embryos were placed individually in a 96-well plate. Measurements were performed at each time point to the same animal subject every time in a blind fashion until day 3 through 4, when the phenotype was apparent. Heart beat count was performed for 15 seconds without anesthetic to avoid any secondary effects that could impact heart rate. Heart rate values were adjusted according to the ANOVA model, for both experiment and time points variability p = 0.000264, F (1,76) = 14.647. dpf, days post fertilization; IF, immunofluorescence; MF20, Myosin Heavy Chain Antibody. (TIFF) [file pbio.3000087.s003.tiff]

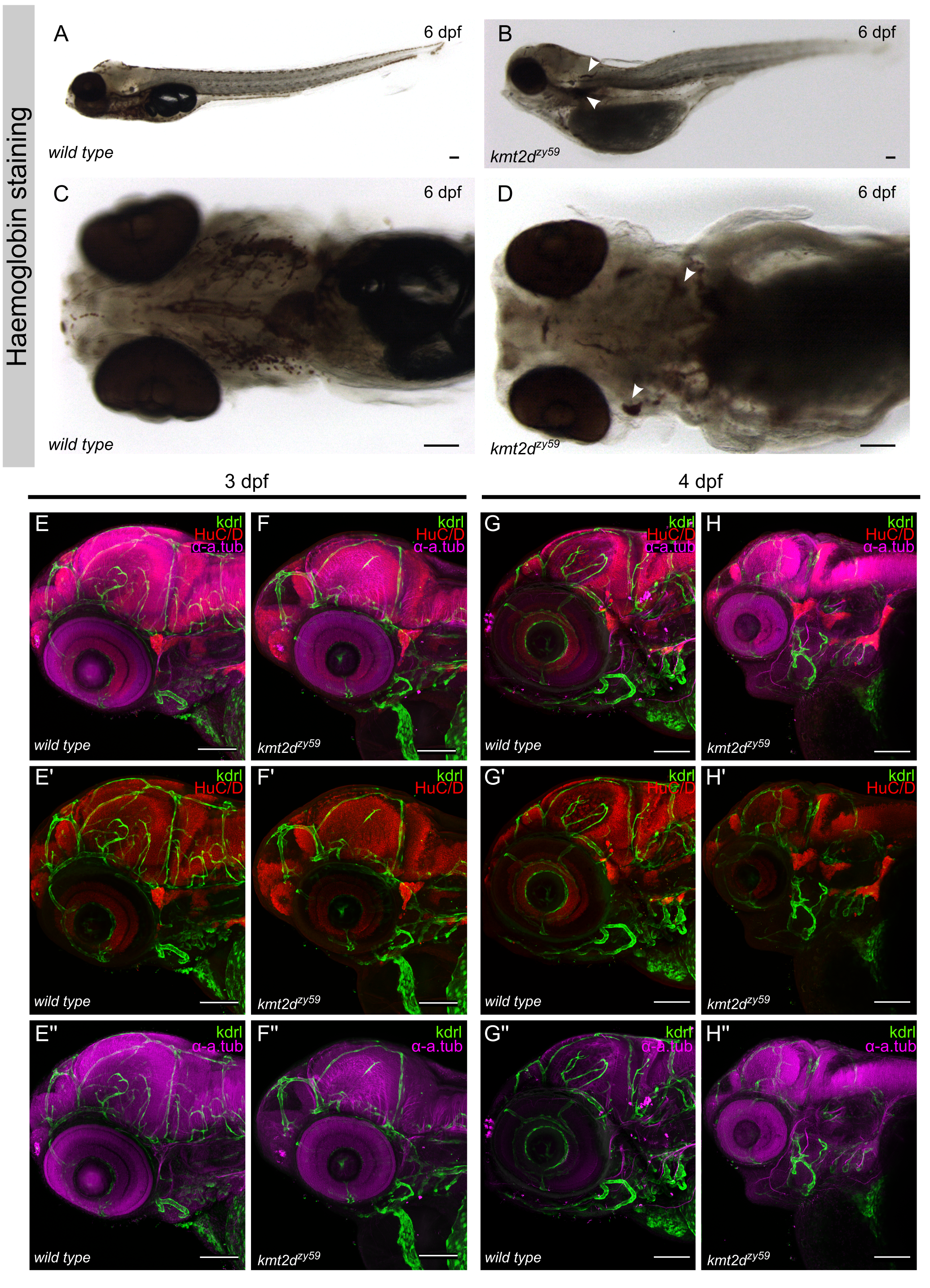

Supplement: S4 Fig — (A–D) o-dianisidine staining for assessing vasculature integrity in kmt2dzy59 and wild-type siblings at 6 dpf. Lateral views (A, B) and cranial-ventral views (C, D) of wild-type sibling (A, C) and kmt2dzy59 mutant (B, D) at 6 dpf. White arrowheads indicate blood aggregates in the region of AA and head. Scale bar = 100 μm. (E–H) Vascular development at 3 dpf and 4 dpf in wild-type sibling versus kmt2dzy59 mutant embryos. Confocal images of cranio-lateral views at 3 dpf (E, F) and 4 dpf (G, H) in wild-type (E–E", G, G") and mutant (F–F", H, H") embryos. IF was performed against GFP, for enhancing Kdrl:GFP transgenic signal, HuC/D and α-acetylated tubulin as context markers. AA, aortic arch; dpf, days post fertilization; GFP, green fluorescent protein; IF, mmunofluorescence; kdrl, kinase insert domain receptor like. (TIFF) [file pbio.3000087.s004.tiff]

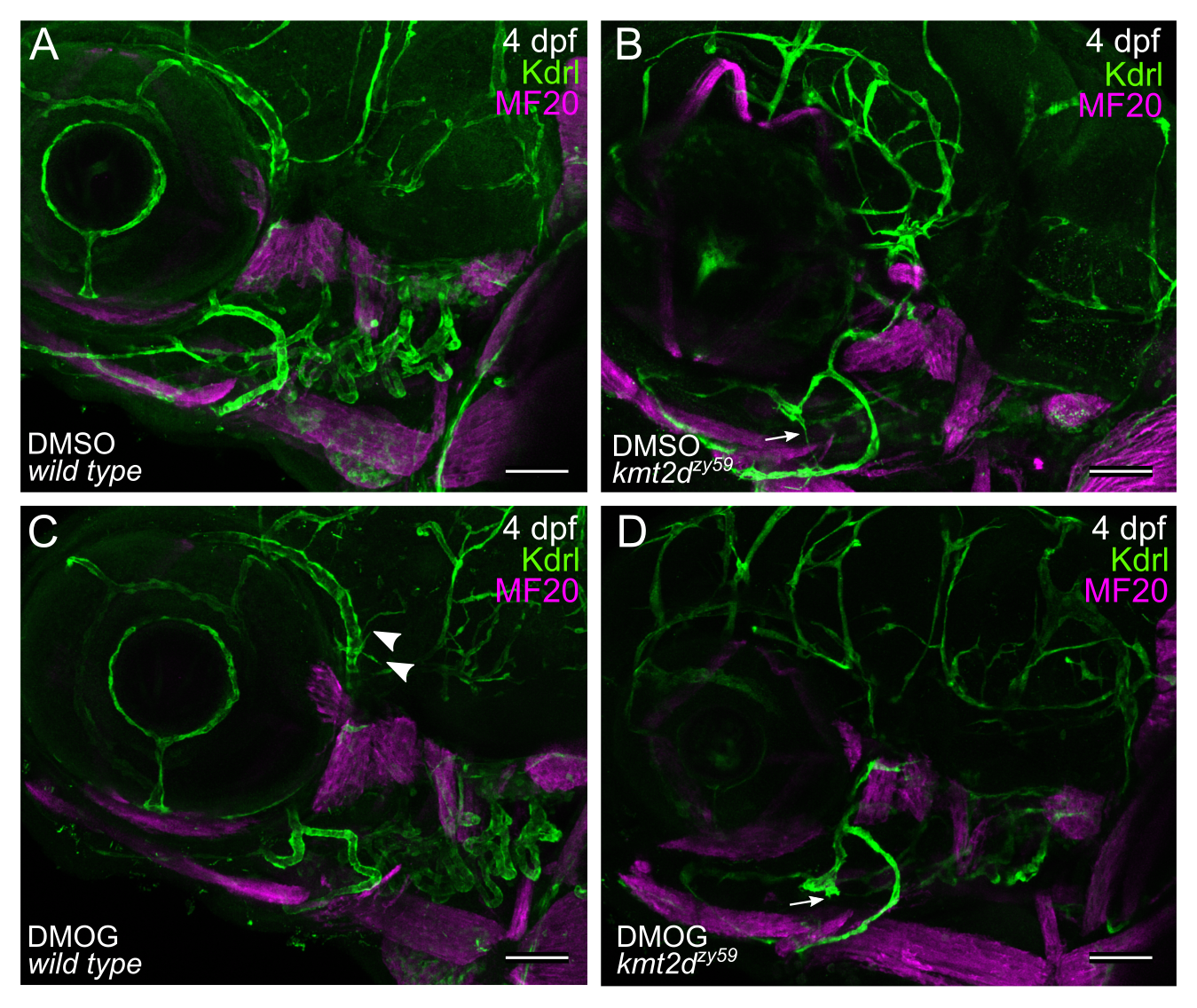

Supplement: S5 Fig — Confocal images show cranial-lateral view of vasculature in wild-type tg(kdrl:GFP) sibling (A) and kmt2dzy59;tg(kdrl:GFP) mutants at 4 dpf. (A–B) DMSO controls for both wild-type sibling and kmt2d mutant. (C–D) DMOG treated embryos. Treatment was performed from 3 to 4 dpf. White arrowheads indicate hypoxia-induced blood vessel sprouting. White arrows (B and D) indicate kmt2d mutation-dependent ectopic blood vessel formation in both DMSO control and DMOG treated embryos. dpf, days post fertilization. (TIFF) [file pbio.3000087.s005.tiff]

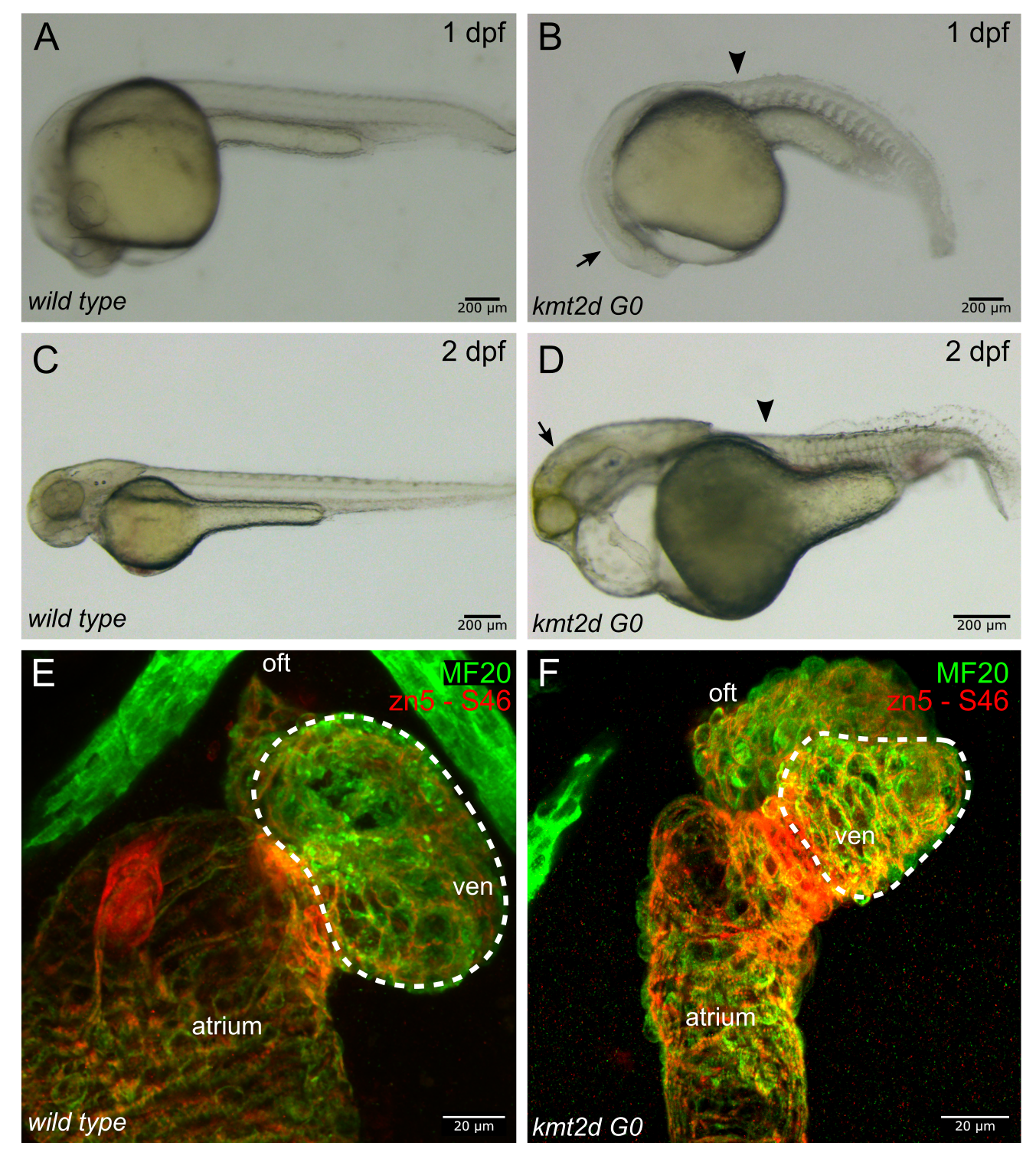

Supplement: S6 Fig — CRISPR/Cas9 injection against kmt2d produces comparable phenotype to the observed in germline mutants (arrows and arrowheads). A, C, E, Noninjected controls. B, D, F, injected embryos. E, F, confocal images of noninjected controls and kmt2d injected embryos. IF was performed for Myosin heavy chain (M20, green), Alcama (zn5, red), and Myosin heavy chain, atrium specific (S46, red) as general myocardium morphology markers. Dashed white line highlights hypoplastic heart as a consequence of mutated kmt2d through CRISPR injection. F0, filial 0; IF, immunofluorescence; kmt2d, Histone-lysine N-methyltransderase 2D; M20, Myosin Heavy Chain Antibody. (TIFF) [file pbio.3000087.s006.tiff]

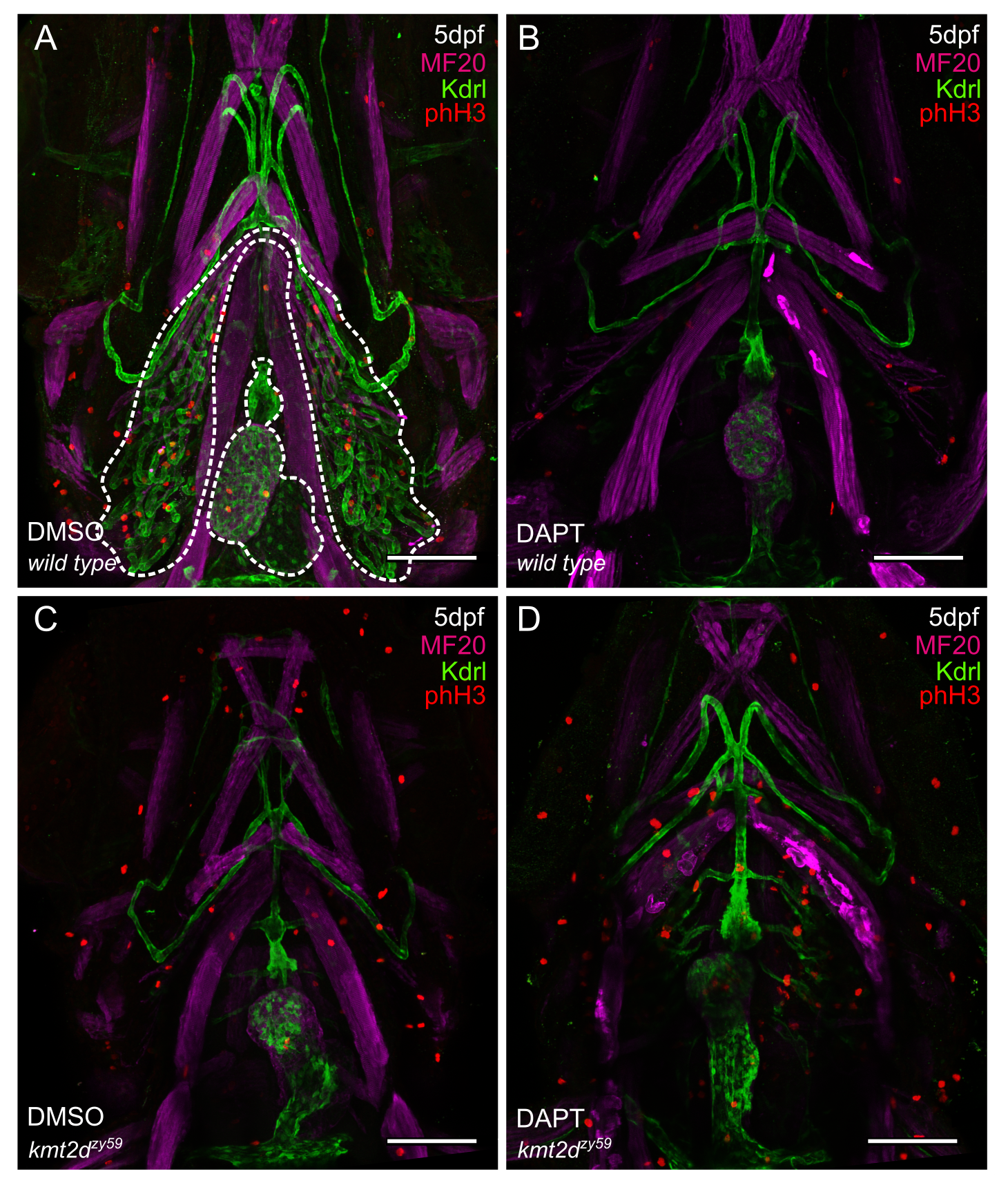

Supplement: S7 Fig — (A–D) Confocal images of wild-type sibling (A, B) and kmt2dzy59 mutant (C, D) embryos at 5 dpf. DMSO as solvent control (A, C) and DAPT for Notch signaling inhibition (B, D) were applied to embryos of indicated genotypes. IF against GFP was performed to enhance Kdrl:GFP trangenic signal (endothelium and endocardium). MF20 (myosin) was use as context marker for muscle. phH3 (cell proliferation) marks mitotic cells. Note the increased phH3 signal in cardiovascular area in kmt2d mutants after DAPT treatment (D). dpf, days post fertilization; GFP, green fluorescent protein; IF, immunofluorescence; kdrl, kinase insert domain receptor like; MF20, Myosin Heavy Chain Antibody; phH3, phospho Histone 3 (TIFF) [file pbio.3000087.s007.tiff]

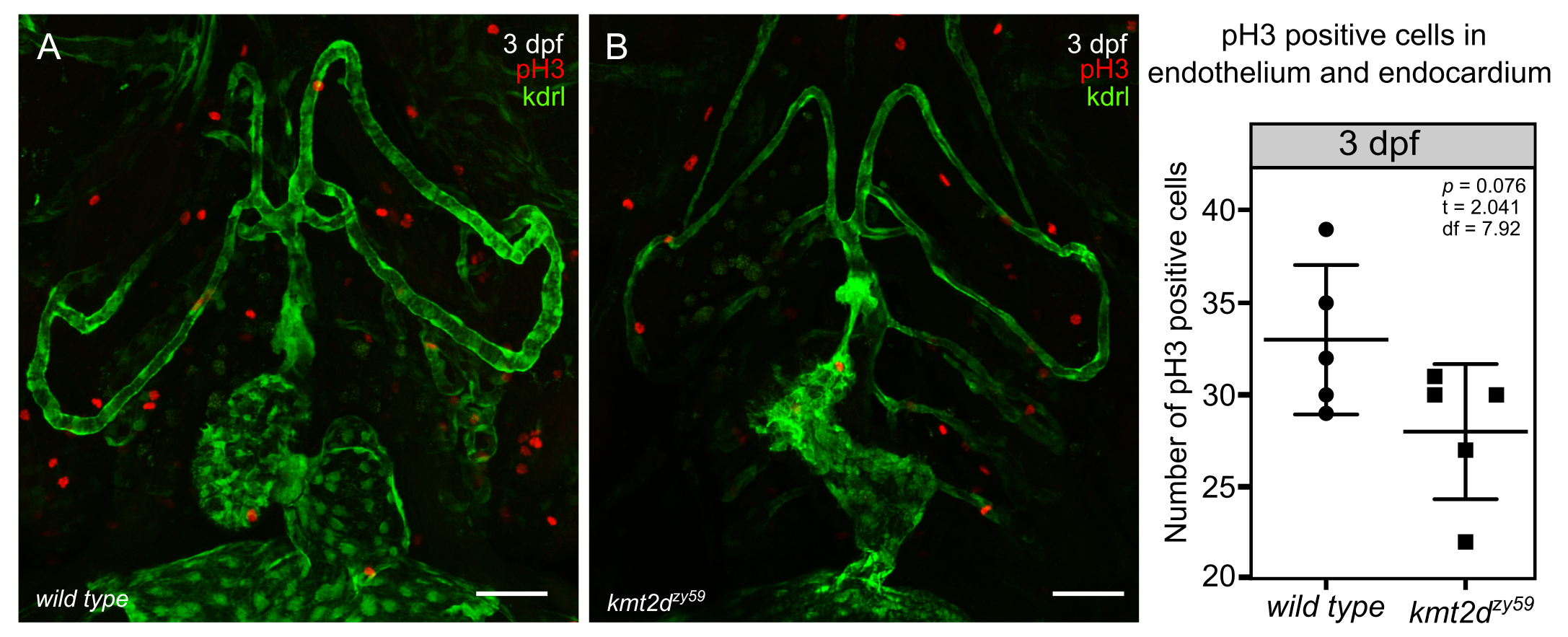

Supplement: S8 Fig — (A–B) Confocal images of wild-type sibling (A) and kmt2dzy59 mutant (B) embryos at 3 dpf. IF against GFP was perform to enhance Kdrl:GFP trangenic signal (endothelium and endocardium). pH3 (cell proliferation) marks mitotic cells. Quantification of pH3 was performed exclusively in endothelial and endocardial cells using Imaris (version 9.2) software. dpf, days post fertilization; GFP, green fluorescent protein; IF, immunofluorescence; kdrl, kinase insert domain receptor like. (TIFF) [file pbio.3000087.s008.tiff]
