# Supplementary material for: Inhibition of Notch signaling rescues cardiovascular development in Kabuki Syndrome
Source: PLoS Biol. 2019 Sep 3;17(9):e3000087. doi: 10.1371/journal.pbio.3000087 (PMC6743796; doi:10.1371/journal.pbio.3000087)
Supplement: S1 Table — Top 50 gene candidate at a 5% FDR (base mean, log2 fold change and p adjusted values are specified). Manual text-mining was performed for each candidate. Categories were established considering available information for cell compartment, biological function, region of expression and mammal orthologs data. FDR, false discovery rate. (DOCX) [file pbio.3000087.s010.docx]

**S1 Table**

| ensembl_gene_id | | external_gene_name | baseMean | log2FoldChange | padj | region_expression | category | source | link |  |
| --- | --- | --- | --- | --- | --- | --- | --- | --- | --- | --- |
| ENSDARG00000018264 | trim101 | | 223.266953 | -3.4205734 | 4.92E-25 | skeletal_muscle | muscle | zfin | <http://zfin.org/ZDB-GENE-040801-100> |  |
| ENSDARG00000039929 | ckmt2b | | 68.3279066 | -1.2735683 | 1.79E-05 | skeletal_muscle | muscle | zfin | <http://zfin.org/ZDB-GENE-040426-1654> |  |
| ENSDARG00000061827 | zbtb4 | | 141.480592 | -1.0032933 | 4.16E-09 | mesoderm | muscle | zfin | <https://zfin.org/ZDB-GENE-100318-5> |  |
| ENSDARG00000075842 | pigt | | 61.6656557 | -0.8932759 | 1.33E-05 | somite | muscle | zfin | <https://zfin.org/ZDB-GENE-090313-46> |  |
| ENSDARG00000039265 | arhgap4a | | 38.0616206 | -1.8567648 | 1.18E-08 | neural_tissue | neural | zfin - OMIN | <http://zfin.org/ZDB-GENE-040426-1229> | <https://omim.org/entry/300023?search=arhgap4&highlight=arhgap4> |
| ENSDARG00000094426 | her4.2 | | 692.157419 | -1.4117474 | 1.23E-12 | neural_tissue | neural | zfin | <http://zfin.org/ZDB-GENE-060815-1> |  |
| ENSDARG00000010347 | acer1 | | 112.087892 | -1.2215564 | 9.54E-07 | neural_tissue | neural | zfin | <http://zfin.org/ZDB-GENE-050417-70> |  |
| ENSDARG00000070770 | her4.3 | | 564.221021 | -1.1805975 | 4.19E-05 | neural_tissue | neural | zfin | <https://zfin.org/ZDB-GENE-081030-7> |  |
| ENSDARG00000037140 | pfkfb1 | | 214.42707 | -0.8844084 | 1.19E-11 | neural_tissue | neural | zfin | <https://zfin.org/ZDB-GENE-030131-5664> |  |
| ENSDARG00000034896 | ldb2b | | 250.609608 | -0.6198509 | 1.33E-05 | neural_tissue | neural | zfin | <https://zfin.org/ZDB-GENE-990415-137> |  |
| ENSDARG00000033533 | CCDC115 | | 524.303483 | -0.4989453 | 3.29E-05 | neural_tissue | neural | zfin - OMIN | <https://zfin.org/ZDB-GENE-050227-20> | <https://omim.org/entry/613734> |
| ENSDARG00000069981 | cspg5a | | 829.926292 | -0.4933447 | 1.04E-05 | neural_tissue | neural | zfin - OMIN | <https://zfin.org/ZDB-GENE-080425-4> | <https://omim.org/entry/606775> |
| ENSDARG00000023174 | fez1 | | 524.997667 | -0.4801264 | 1.54E-06 | neural_tissue | neural | zfin | <https://zfin.org/ZDB-GENE-040426-2723> |  |
| ENSDARG00000076081 | sncgb | | 353.493441 | -0.4567478 | 2.63E-05 | neural_tissue | neural | zfin | <https://zfin.org/ZDB-GENE-050522-235> |  |
| ENSDARG00000030547 | rnd1 | | 448.576717 | 0.90929143 | 6.37E-08 | neural_tissue | neural | zfin | <https://zfin.org/ZDB-GENE-040630-6> |  |
| ENSDARG00000089549 | BAALC | | 88.3687299 | 1.28939661 | 1.03E-09 | neural_tissue | neural | zfin - OMIN | <https://zfin.org/ZDB-GENE-081022-24> | <https://omim.org/entry/606602> |
| ENSDARG00000090472 | ttll10 | | 138.533229 | 1.96887323 | 7.30E-23 | neural_tissue | neural | zfin | <https://zfin.org/ZDB-GENE-081104-350> |  |
| ENSDARG00000091235 | CABZ01015525.1 | | 47.5085214 | -3.4152642 | 4.01E-05 | vascular_tissue | neural and cardiovascular | Institute of Cardiovascular Regeneration, Goethe University Frankfurt | <http://angiogenes.uni-frankfurt.de/transcript?search=&rows_per_page_int=25&page_number=546> |  |
| ENSDARG00000054817 | ppp1r14c | | 5.54292909 | -5.4382032 | 2.69E-05 | neural_cardiac_tissue | neural and cardiovascular | OMIN | <https://omim.org/entry/613242> |  |
| ENSDARG00000020788 | sla2 | | 23.4693679 | -2.3799544 | 9.45E-11 | immune_system | neural and cardiovascular | zfin - OMIN | <http://zfin.org/ZDB-GENE-080204-98> | <https://omim.org/entry/606577> |
| ENSDARG00000089920 | CU571255.1 | | 47.1329575 | -1.2207048 | 5.22E-06 | cardiac_skeletal_muscle | neural and cardiovascular | zfin | <https://zfin.org/ZDB-GENE-041111-277> |  |
| ENSDARG00000070486 | rbp7b | | 985.092104 | -0.9525541 | 1.57E-06 | neural_cardiac_muscle_tissue | neural and cardiovascular | zfin | <https://zfin.org/ZDB-GENE-081022-134> |  |
| ENSDARG00000002644 | rgs5a | | 164.568151 | -0.6718394 | 2.06E-06 | vascular_tissue | neural and cardiovascular | zfin | <https://zfin.org/ZDB-GENE-030131-7570> |  |
| ENSDARG00000056831 | gng2 | | 366.318584 | -0.5695759 | 1.79E-05 | neural_vascular_tissue | neural and cardiovascular | zfin | <https://zfin.org/ZDB-GENE-050417-59> |  |
| ENSDARG00000086826 | sult6b1 | | 1349.51741 | -0.5633785 | 1.35E-12 | neural_vascular_tissue | neural and cardiovascular | zfin | <https://zfin.org/ZDB-GENE-050417-228> |  |
| ENSDARG00000056499 | ca6 | | 1780.87737 | -0.5397003 | 2.46E-09 | neural_vascular_tissue | neural and cardiovascular | zfin | <https://zfin.org/ZDB-GENE-030131-7091> |  |
| ENSDARG00000013855 | slc12a3 | | 493.854667 | -0.5100772 | 5.31E-06 | neural_cardiac_gut | neural and cardiovascular | zfin | <https://zfin.org/ZDB-GENE-030131-9505> |  |
| ENSDARG00000020785 | lama4 | | 1142.44679 | -0.4982175 | 3.09E-05 | cardiac_vascular_tissue | neural and cardiovascular | zfin | <https://zfin.org/ZDB-GENE-040724-213> |  |
| ENSDARG00000063538 | kalrnb | | 505.638601 | -0.4433807 | 1.31E-05 | blood | neural and cardiovascular | zfin | <https://zfin.org/ZDB-GENE-060421-7244> |  |
| ENSDARG00000056929 | kdm6bb | | 1769.32634 | -0.4381307 | 1.16E-06 | neural_cardiac_vascular_tissue | neural and cardiovascular | zfin | <https://zfin.org/ZDB-GENE-040724-166> |  |
| ENSDARG00000056075 | rca2.1 | | 1170.46545 | -0.3794535 | 1.24E-06 | neural_cardiac_gut | neural and cardiovascular | zfin | <https://zfin.org/ZDB-GENE-050320-60> |  |
| ENSDARG00000018404 | krt18 | | 16821.7388 | 0.53661864 | 3.32E-05 | cardiac_vascular__tissue_pharyngeal_arch | neural and cardiovascular | zfin | <https://zfin.org/ZDB-GENE-030411-6> |  |
| ENSDARG00000095512 | rca2.2 | | 43.3431095 | 1.47416929 | 3.04E-09 | neural_cardiac_liver_tissue | neural and cardiovascular | zfin | <https://zfin.org/ZDB-GENE-060503-646> |  |
| ENSDARG00000002945 | bgnb | | 49.9919093 | 1.76077257 | 1.11E-09 | neural_cardiac_vascular_tissue | neural and cardiovascular | zfin - OMIN | <https://zfin.org/ZDB-GENE-040426-21> | <https://omim.org/entry/300989> |
| ENSDARG00000004836 | dnajc5ab | | 1423.26869 | 0.39387467 | 8.17E-09 | neural_testis_tissue | neural and reproductive | zfin - OMIN | <https://zfin.org/ZDB-GENE-081021-2> | <https://omim.org/entry/611203?search=dnajc5&highlight=dnajc5> |
| ENSDARG00000056515 | spsb1 | | 570.752435 | 0.53790646 | 2.71E-08 | neural_muscle_ovary_testis | neural and reproductive | zfin | <https://zfin.org/ZDB-GENE-030131-6122> |  |
| ENSDARG00000005464 | dnase1l3 | | 710.501878 | -0.7709107 | 1.53E-05 | neural_tissue_blood_pharyngeal_arch | neural, blood and pharyngeal arches | zfin | <https://zfin.org/ZDB-GENE-040808-35> |  |
| ENSDARG00000089489 | CR762484.3 | | 272.882325 | -0.6017719 | 3.93E-10 | neural_tissue_hematopoietic | neural, blood and pharyngeal arches | zfin | <https://zfin.org/ZDB-GENE-000804-1> |  |
| ENSDARG00000057863 | dnmt5 | | 924.315763 | 0.36278089 | 1.31E-05 | neural_tissue_blood_pharyngeal_arch | neural, blood and pharyngeal arches | zfin | <https://zfin.org/ZDB-GENE-050314-2> |  |
| ENSDARG00000057426 | oard1 | | 162.83978 | -2.2705516 | 5.41E-39 |  | other | zfin | <http://zfin.org/ZDB-GENE-050522-480> |  |
| ENSDARG00000052336 | ociad2 | | 98.6553524 | -1.0976163 | 3.95E-09 |  | other | zfin | <https://zfin.org/ZDB-GENE-041014-253> |  |
| ENSDARG00000034714 | esyt1a | | 381.761581 | -0.7463954 | 8.37E-08 | spleen_adipose_tissue | other | zfin - OMIN | <https://zfin.org/ZDB-GENE-090311-55> | <https://omim.org/entry/616670> |
| ENSDARG00000070657 | pa2g4b | | 7279.50801 | -0.5600499 | 1.64E-06 |  | other | zfin | <https://zfin.org/ZDB-GENE-030131-2182> |  |
| ENSDARG00000037071 | rps26 | | 8956.16534 | 0.39521037 | 4.65E-06 |  | other | zfin | <https://zfin.org/ZDB-GENE-030131-8606> |  |
| ENSDARG00000056122 | gdi1 | | 2743.84085 | 0.58411227 | 4.41E-12 |  | other | zfin | <https://zfin.org/ZDB-GENE-050522-504> |  |
| ENSDARG00000017773 | slc16a12a | | 153.26096 | 0.90229925 | 1.64E-06 |  | other | zfin | <https://zfin.org/ZDB-GENE-080721-24> |  |
