# Supplementary material for: Inhibition of Notch signaling rescues cardiovascular development in Kabuki Syndrome
Source: PLoS Biol. 2019 Sep 3;17(9):e3000087. doi: 10.1371/journal.pbio.3000087 (PMC6743796; doi:10.1371/journal.pbio.3000087)
Supplement: S3 Table — (DOCX) [file pbio.3000087.s012.docx]

**Key Resources Table**

| REAGENT or RESOURCE | SOURCE | IDENTIFIER |
| --- | --- | --- |
| Antibodies | | |
| Mouse IgG2b monoclonal anti myosin, sarcomere (MHC) | DSHB | MF20 |
| Mouse IgG1 monoclonal anti alcama | ZIRC | Zn-5 |
| Rabbit polyclonal anti active-Caspase-3 | Abcam | ab13847 |
| Rabbit polyclonal anti Kmt2d | Antibody Verify | AAS14579C |
| Chicken polyclonal anti GFP | Aves Lab | GFP-1020 |
| Rabbit polyclonal anti RBPJ | ABclonal | A5675 |
| Goat Secondary antibodies against primary. All from the Alexa family. | ThermoFisher Scientific | A-21144/A-11008/A-21130/A-21124 |
| Bacterial and Virus Strains | | |
| Subcloning Efficiency DH5α Competent Cells | ThermoFisher Scientific | 18265017 |
| Chemicals, Peptides, and Recombinant Proteins | | |
| N-Phenylthiourea – (PTU). Used for avoiding pigment formation in *in vivo* time-lapse experiment | Sigma-Aldrich | P7629 |
| Ethyl 3-aminobenzoate methanesulfonate (MS-222/Tricaine), pH 7 | Sigma-Aldrich | A5040 |
| HIF-Hydroxylase Inhibitor, DMOG. Used in hypoxia experiments | Calbiochem | CAS 89464-63-1 |
| DAPT (GSI-IX). Used in Notch inhibition experiments | SelleckChem | S2215 |
| Dimethyl sulfoxide (DMSO). Solvent control | Sigma-Aldrich | D2650-5X5ML |
| Agarose II, for biotechnology | vwr | 97062-400 |
| Goat serum | Sigma-Aldrich | G9023 |
| Alcian Blue Solution | Sigma-Aldrich | B8438 |
| Red Alizarin | MG scientific | AL-200-25 |
| o-Dianisidine | Sigma-Aldrich | D9143 |
| TrypLE | Gibco | 12604 |
| Fetal Bovine Serum (FBS) | Gibco | 26041 |
| TRI Reagent | Zymo Research | R2050 |
| Critical Commercial Assays | | |
| Quick-DNA/RNA. Extraction Kit | Zymo Research | D7005 |
| Alt-R™ S.p. Cas9 Nuclease 3NLS | IDT | 1074181 |
| Zymo mini Quick-RNA kit | Zymo Research | R1054 |
| BioRad iScript 5X master mix | Bio-Rad | 1708840 |
| Deposited Data | | |
|  |  |  |
| Experimental Models: Organisms/Strains | | |
| *tg(kdrl*:*EGFP)^la116^* | NA | (Mathias *et al.*, 2010) |
| *Tg(EPV.Tp1-Mmu.Hbb:EGFP)^um14^* | NA | (Nicoli *et al.*, 2010) |
| *Kmt2d^zy59^* | NA | This paper |
| *Kmt2d^zy59^; tg(kdrl*:*EGFP)^la116^* | NA | This paper |
| Oligonucleotides | | |
| *kmt2d* guide RNA 5’-GTATTGACTGTGGCATGCGA-3’ |  | This paper |
| *kmt2d* HRMA primer Forward 5’-GTTTTAGCGGTGCCGTGTG-3’ | NA | This paper |
| *kmt2d* HRMA primer Reverse 5’- CCACTGTTCAGAGCCAGGAAG-3’ | NA | This paper |
| *kmt2d* Sequencing primer Forward 5’- GGAAAAGTACACTTTAGAAAACAGC-3’ | NA | This paper |
| *kmt2d* Sequencing primer Reverse 5’- AGCTTGTACAGAAGTTTGGCAA-3’ | NA | This paper |
| *notch1b* Forward 5’-GACATGCAGAACAATAAGGAGG-3’ | NA | This paper |
| *notch1b* Reverse 5’- cagaagaactttagccgtctc-3’ | NA | This paper |
| *rbpja* Forward 5’- GATAATTCAGTTCCAGGCCAC-3’ | NA | This paper |
| *rbpja* Reverse 5’- ATGATTGTCCATGATGCACC-3’ | NA | This paper |
| *hes1* Forward 5’- GGGATGGATTGTGAGGTGAATA-3’ | NA | This paper |
| *hes1* Reverse 5’- AGCCAGCACAGGAGAAATAC-3’ | NA | This paper |
| *elfα* Forward 5’-CTTCTCAGGCTGACTGTGC-3’ | NA | (McCurley and Callard, 2008) |
| *elfα* Reverse 5’-CCGCTAGCATTACCCTCC-3’ | NA | (McCurley and Callard, 2008) |
| Recombinant DNA | | |
| pKMT-E5-S264 – guide RNA for *kmt2d* | NA | This paper |
| Software and Algorithms | | |
| Zen Black | Zeiss | https://www.zeiss.com/microscopy/us/downloads/zen.html |
| FIJI - ImageJ (version 2.0.0-rc-68/1.59g) | NIH(Schindelin *et al.*, 2012) | https://downloads.imagej.net/fiji/ |
| Imaris 9.2 | Bitplane | http://www.bitplane.com/ |
| FlowJo version 9 | FlowJo LLC | http://docs.flowjo.com/vx/ |
| Inkscape 0.92 | Free Software Foundation, Inc | https://inkscape.org |
